# Supplementary material for: Improving the Efficacy of EGFR Inhibitors by Topical Treatment of Cutaneous Squamous Cell Carcinoma with miR-634 Ointment
Source: Mol Ther Oncolytics. 2020 Oct 22;19:294–307. doi: 10.1016/j.omto.2020.10.009 (PMC7695908; doi:10.1016/j.omto.2020.10.009)

OMTO, Volume *19*

Supplemental Information

Improving the Efficacy of EGFR Inhibitors by Topical Treatment of Cutaneous Squamous Cell Carcinoma with *miR-634* Ointment

Jun Inoue, Kyoko Fujiwara, Hidetoshi Hamamoto, Katsunori Kobayashi, and Johji Inazawa

Figure S1

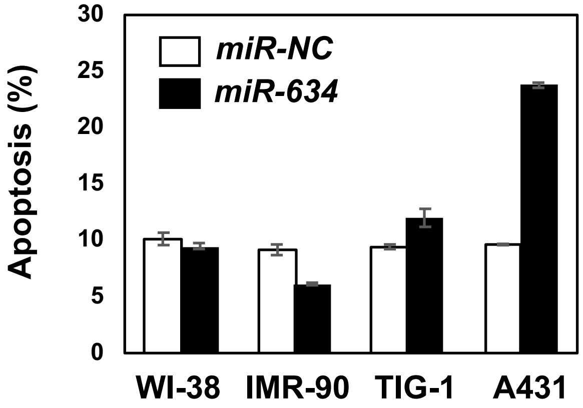

FACS analysis of the apoptotic cell population

FACS analysis was performed at 3 days after transfection. Cells were collected and stained with Annexin V and propidium iodide (PI). Cell population analysis was performed using an Accuri Flow Cytometer. The percentages of apoptotic cells are indicated in each graph. Error bars indicate SD of triplicate experiments.

Figure S2

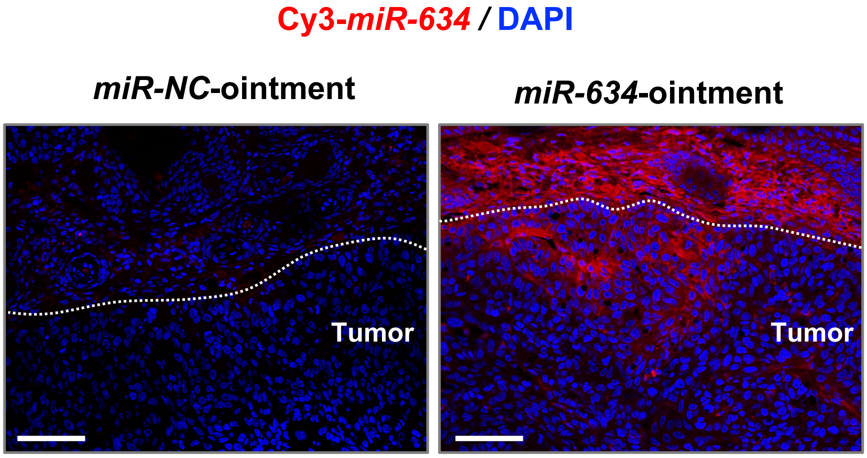

Fluorescence images of frozen subcutaneous tumor sections.
Cy-3-labelled *miR-634* ointment was topically applied to subcutaneous A431 xenograft tumors in mice. At 1 h after application, the tumors were resected, and frozen sections were prepared. Dotted lines indicate the border between tumor tissue and mouse skin. Cy3-*miR-634*, red. DAPI, blue. Scale bar, 100 μm.

Figure S3

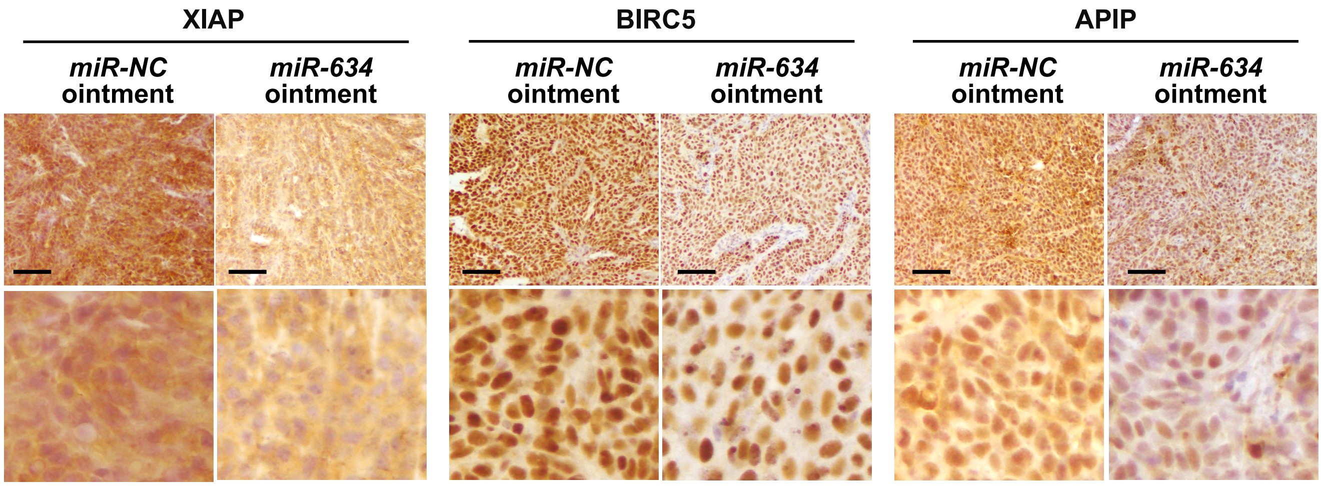

Immunohistochemistry (IHC) analysis of XIAP, BIRC5, and APIP in tumors from mice treated with *miR-NC* ointment or *miR-634* ointment.
Lower panel; enlarged images. Bar; 100 μm.

Figure S4

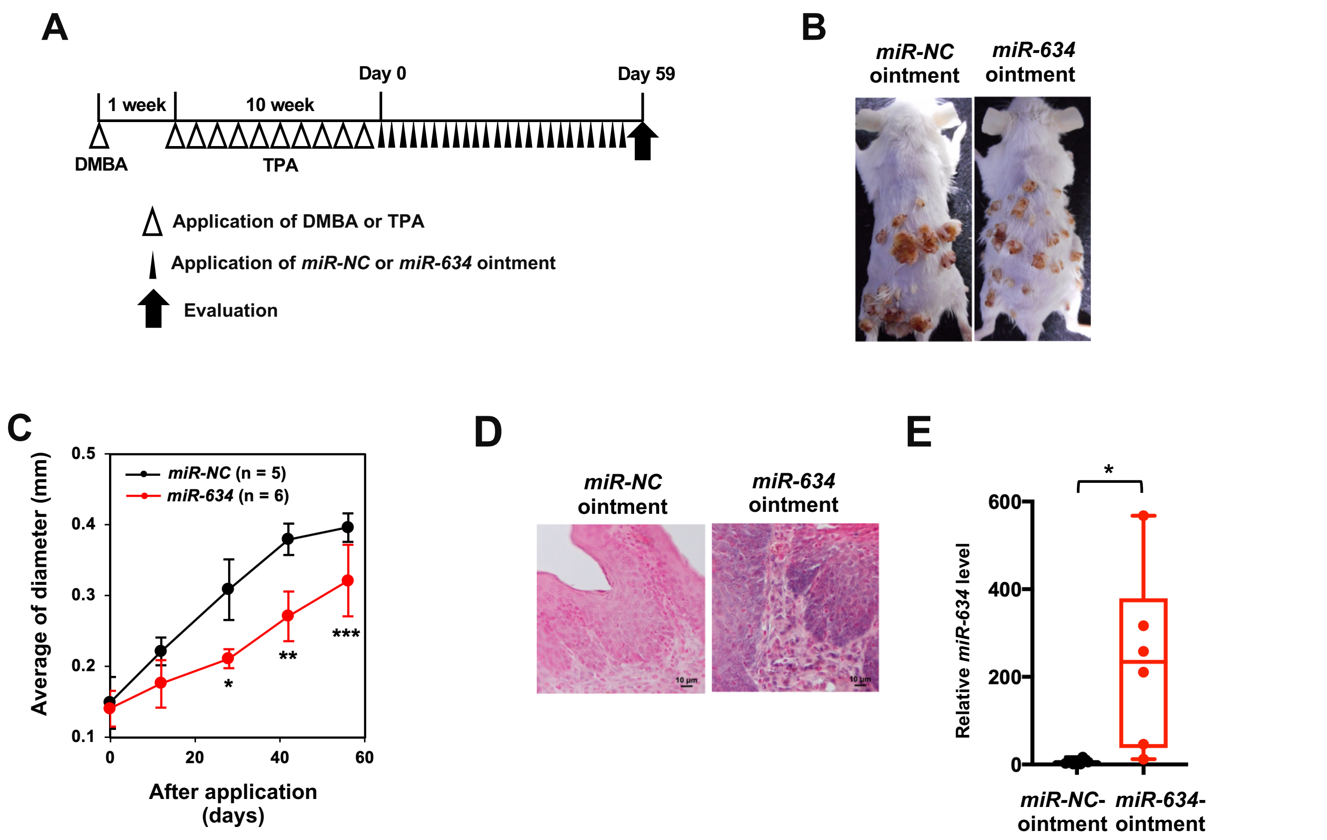

Antitumor effect of *miR-634* ointment in the DMPA/TPA-induced skin papilloma mouse model

A. Experimental schedule for the induction of papilloma and treatment. *miR-NC* ointment or *miR-634* ointment was topically applied twice per week. At 59 days after the first application, the mice were sacrificed, and papillomas were evaluated.
B. Representative images of papilloma after 59 day of treatment.
C. The average diameter was measured in mice treated with *miR-NC* ointment (n = 5) or *miR-634* ointment (n = 6). Error bars indicate the SD. Data are presented as mean ± SD. *P*-values were calculated using the two-sided Student's *t*-test (**p* = 0.0005 at 28 days, ***p* = 0.00019 at 42 days, and ****p* = 0.0013 at 59 days).
D. *In situ* hybridization (ISH) analysis of *miR-634* in resected papilloma. Representative ISH images in papillomas from mice treated with *miR-NC* ointment or *miR-634* ointment. The *miR-634*-specific probe appears purple in the cytoplasm, and the nucleus was counterstained with nuclear fast red. Scale bars, 10 μm.
E. qRT-PCR expression analysis of *miR-634* in resected papillomas. *miR-634* expression levels in papillomas from mice treated with *miR-NC* ointment (n = 5) or *miR-634* ointment (n = 6) were measured by qRT-PCR, and the results are presented in the box plot. *P*-values were calculated using the two-sided Student's *t*-test (**p* = 0.019).

Figure S5

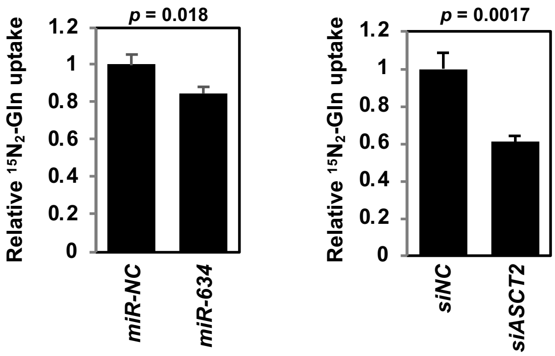

Glutamine uptake assays in *miR-634*-overexpressing cells and *ASCT2*-inhibited cells

A431 cells were transfected with *miR-NC* or *miR-634* (left) and siRNA-*NC* (*siNC*) or siRNA-*ASCT2* (*siASCT2*). After 3 days of transfection, $^{15}N_2$-labelled glutamine was added to the medium, and metabolites were extracted after 2 h (for miRNAs) or 15 min (for siRNAs). Intracellular $^{15}N_2$-labelled glutamine levels per cells were measured using LC/MS. Data are presented as mean ± SD. *P*-values were calculated using the two-sided Student's *t*-test.

Figure S6

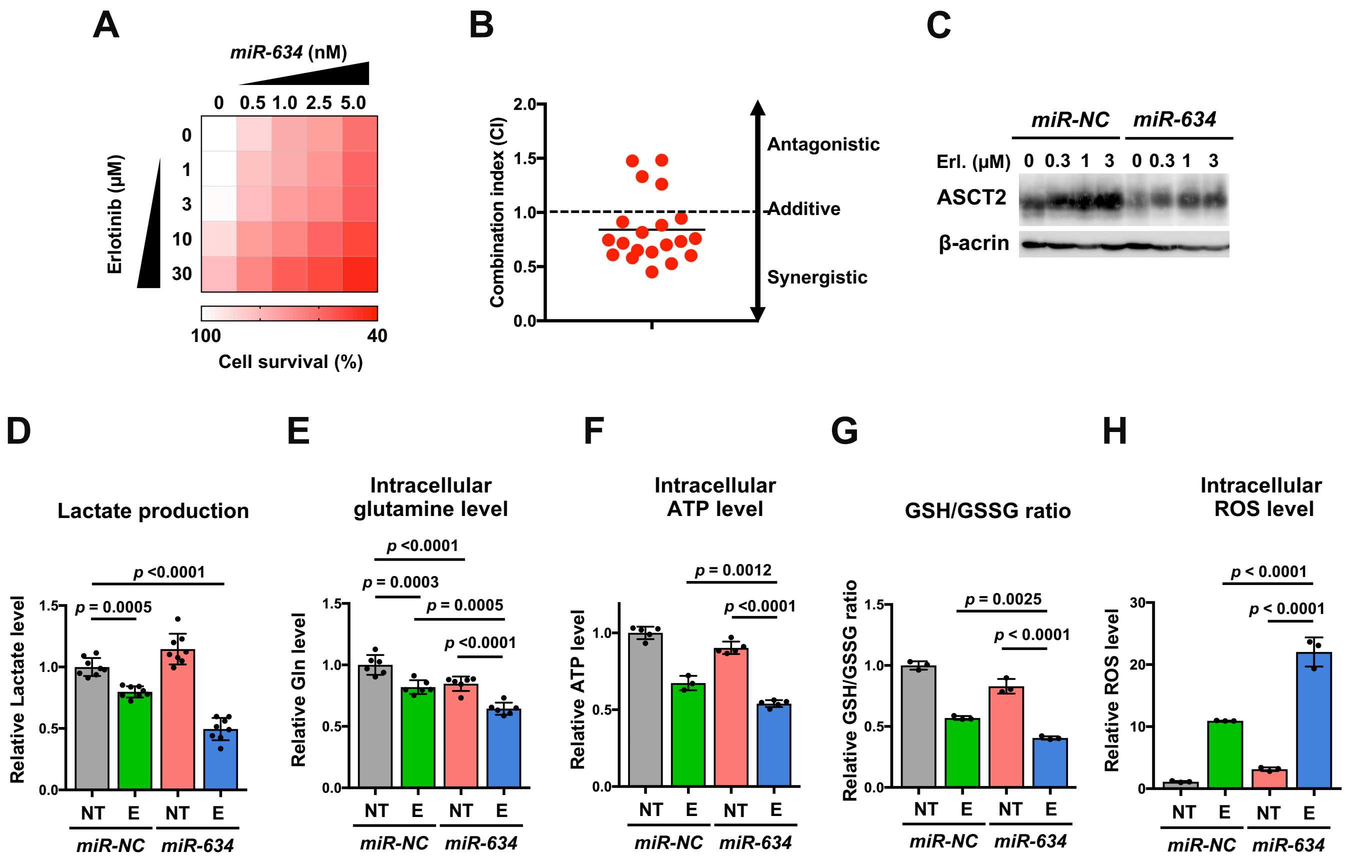

Augmentation of erlotinib-induced cytotoxicity by *miR-634*

A. Cell survival assay. A431 cells were transfected with increasing doses of *miR-634* (1.0 to 10 nM) of *miR-634* and simultaneously treated with erlotinib (1 to 30 μM). After 24 h of erlotinib treatment, the cell survival rate was assessed by crystal violet (CV) staining, and the results are reported as the relative rate compared with non-transfected and non-treated cells.

B. Combination index for A431 cells treated with erlotinib and *miR-634*.

C. Western blotting analysis of ASCT2 and apoptosis markers. Cell lysates were separated by SDS-PAGE and immunoreacted with the indicated antibodies.

D-H. Metabolite analysis of A431 cells treated with erlotinib (E) and/or *miR-634*. A431 cells were transfected with 10 nM of *miR-NC* or *miR-634* and then treated with erlotinib (3 μM) 24 h later. After 24 h of erlotinib treatment, lactate production (d), intracellular glutamine levels (e), intracellular ATP levels (f), the GSH/GSSG ratio (g), and intracellular ROS levels (h) were measured as described in the materials and methods. Data are presented as mean ± SD. *P*-values were calculated using two-way ANOVA.

Figure S7

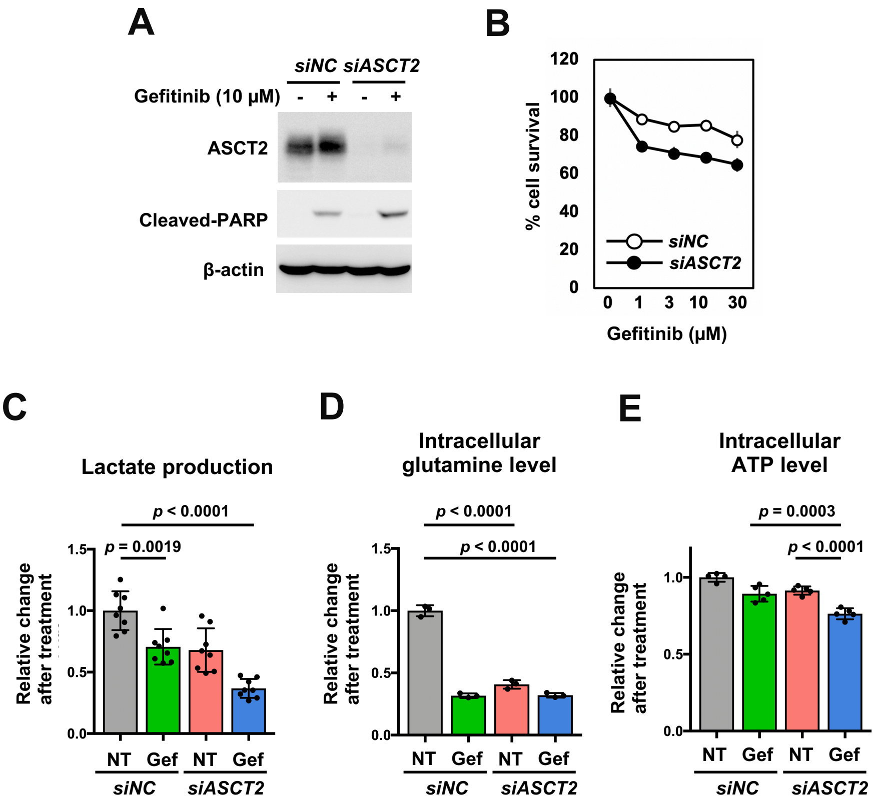

Augmentation of gefitinib-induced cytotoxicity by *ASCT2* knockdown

A. Western blotting analysis of ASCT2 and cleaved PARP. Cell lysates were separated by SDS-PAGE and immunoreacted with the indicated antibodies. Relative expression levels of target genes.

B. Cell survival assay. A431 cells were transfected with 10 nM NC siRNA (*siNC*) or *ASCT2* siRNA (*siASCT2*) and simultaneously treated with gefitinib (1 to 30 μM). After 24 h of gefitinib treatment, the cell survival rate was assessed by crystal violet (CV) staining, and the results are reported as the relative rate compared with non-transfected and non-treated cells.

C-E. Metabolite analysis of A431 cells treated with gefitinib (Gef) and/or *miR-634*. A431 cells were transfected with 10 nM *miR-NC* or *miR-634* and then treated with gefitinib (10 μM) 24 h later. After 24 h of gefitinib treatment, lactate production (c), intracellular glutamine levels (d), and ATP production (e) were measured as described in the materials and methods. Data are presented as mean ± SD. *P*-values were calculated using two-way ANOVA.

Table S1
List of 428 genes downregulated at the protein level in miR-634-overexpressing cells

Table S2
List of 110 genes commonly overlapped from two analyses that were defined as genes directly targeted by miR-634 in A431 cells

Table S3
GO analysis of 110 candidate genes for miR-634 targets

Complete unedited images for western blots

Figure 1B

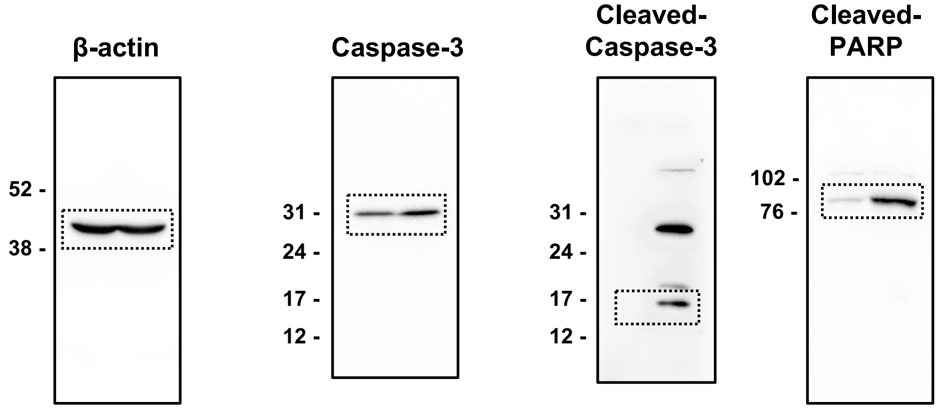

Figure 1D

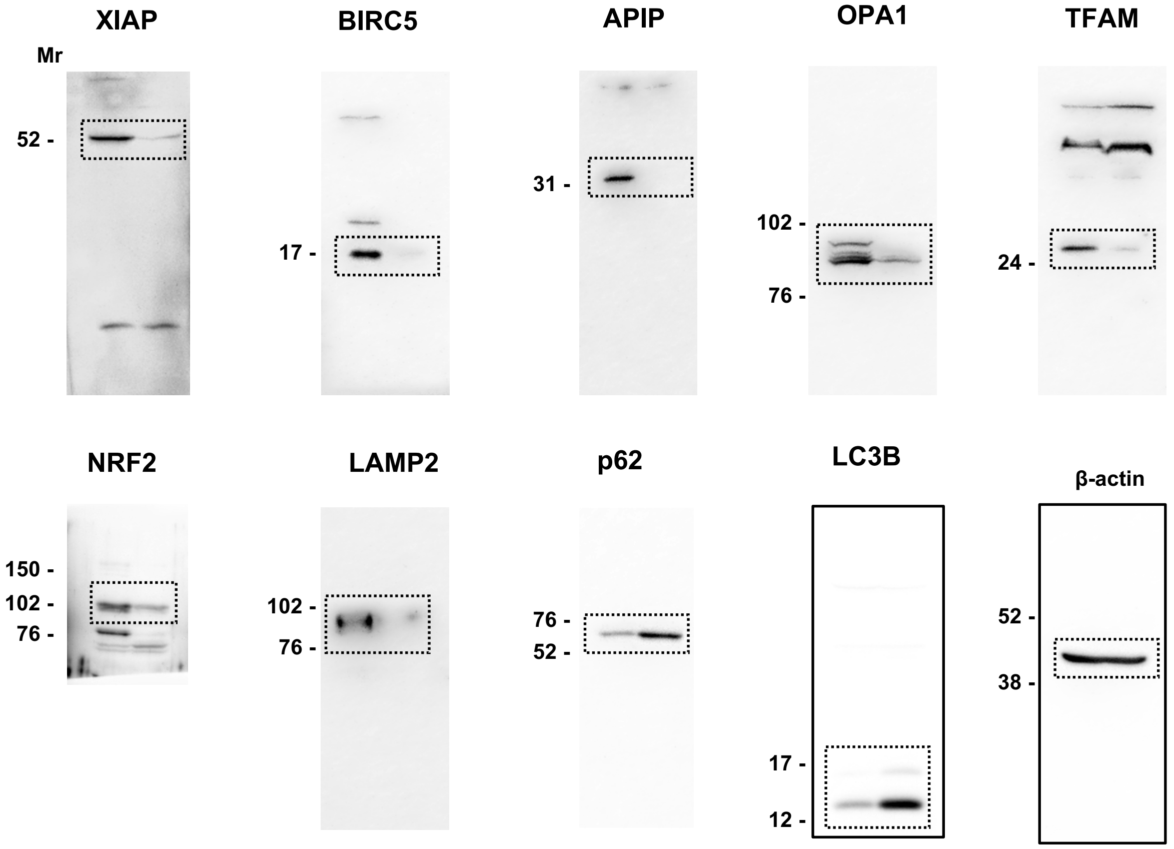

Complete unedited images for western blots (contined)

Figure 1I

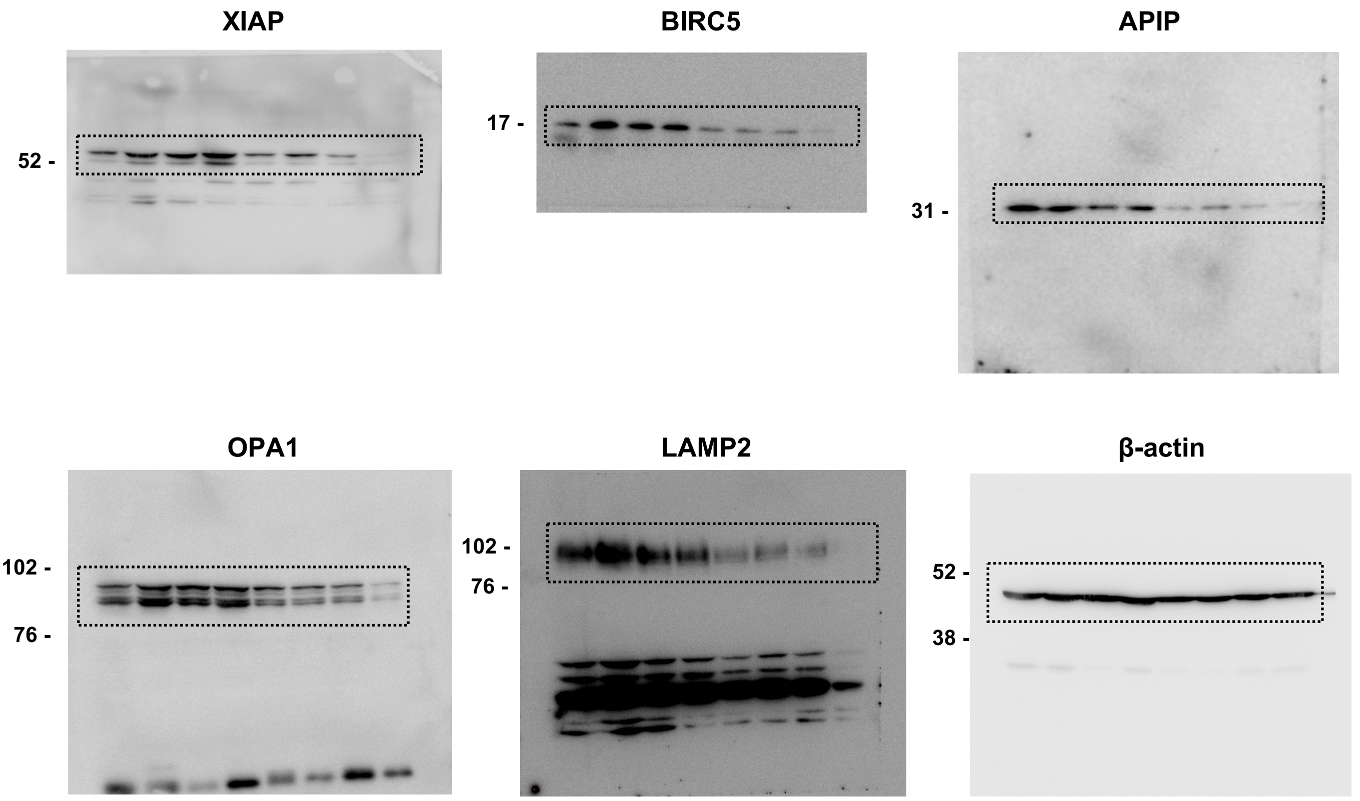

Figure 3

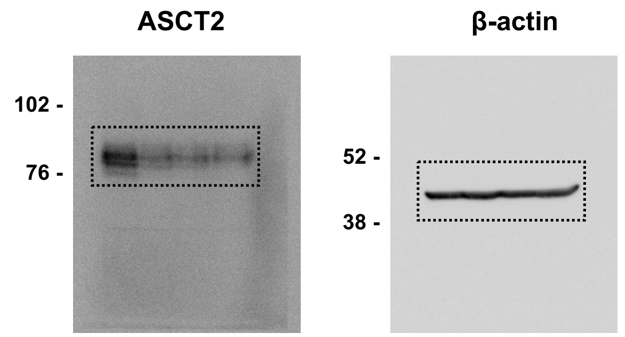

Figure 3F

Figure 3F

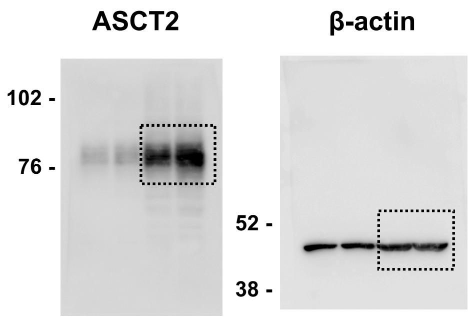

Figure 3G

Figure 3G

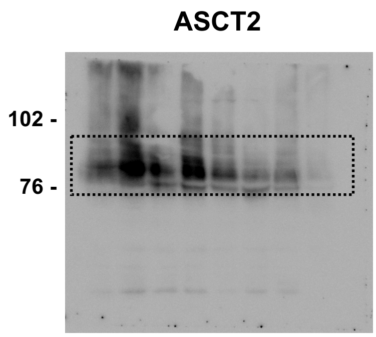

Figure 3I

Complete unedited images for western blots (contined)

Figure 4D

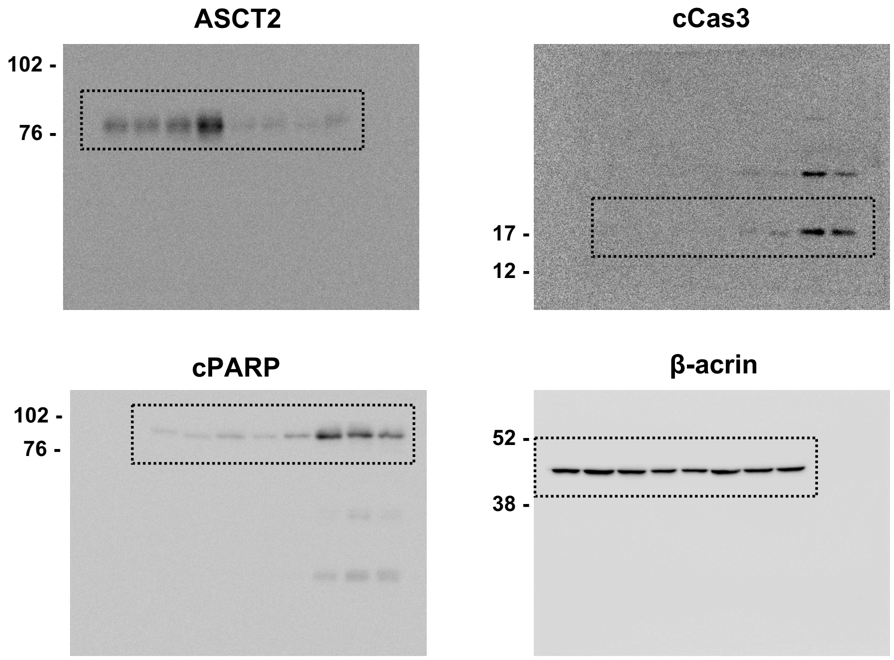

Figure S6C

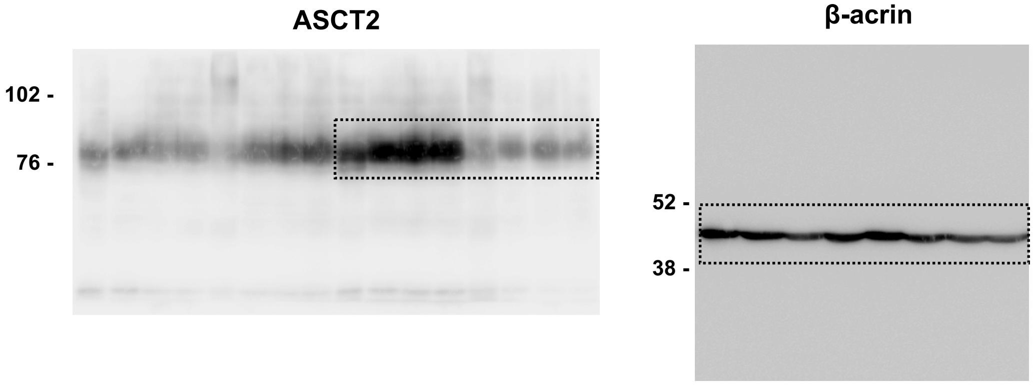

Figure S7A

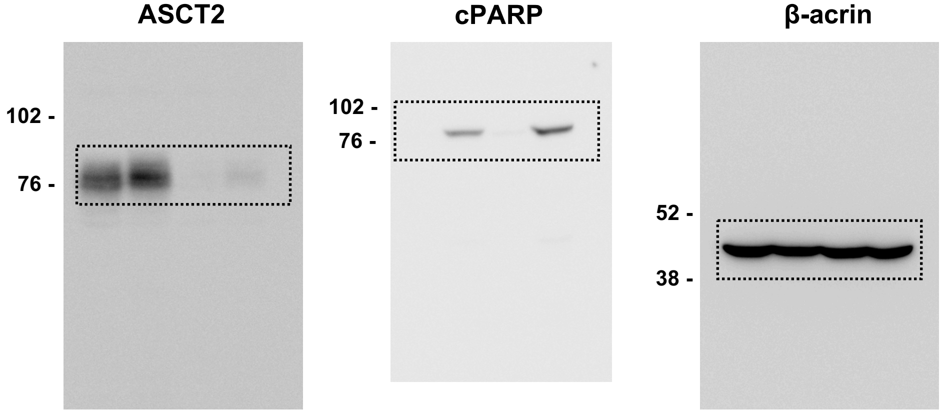

Supplement: Document S1. Figures S1–S7 [file mmc1.pdf]
