# Supplementary material for: Improving the Efficacy of EGFR Inhibitors by Topical Treatment of Cutaneous Squamous Cell Carcinoma with miR-634 Ointment
Source: Mol Ther Oncolytics. 2020 Oct 22;19:294–307. doi: 10.1016/j.omto.2020.10.009 (PMC7695908; doi:10.1016/j.omto.2020.10.009)

Molecular Therapy
Oncolytics
Original Article

Improving the Efficacy of EGFR Inhibitors by Topical Treatment of Cutaneous Squamous Cell Carcinoma with *miR-634* Ointment

Jun Inoue,[1] Kyoko Fujiwara,[2] Hidetoshi Hamamoto,[3] Katsunori Kobayashi,[3] and Johji Inazawa[1,4]

[1]Department of Molecular Cytogenetics, Medical Research Institute, Tokyo Medical and Dental University (TMDU), Tokyo, Japan; [2]Department of Anatomy, Nihon University School of Dentistry, Tokyo, Japan; [3]MEDRx Co., Ltd., Kagawa, Japan; [4]Bioresource Research Center, Tokyo Medical and Dental University (TMDU), Tokyo, Japan

For cutaneous squamous cell carcinoma (cSCC), topical treatment is an essential option for patients who are not candidates for, or who refuse, surgery. Epidermal growth factor receptor (EGFR) plays a key role in the development of cSCC, but EGFR tyrosine kinase inhibitors (TKIs), such as gefitinib, have shown only partial clinical benefit in this disease. Thus, there is an unmet need to develop novel strategies for improving the efficacy of TKIs in cSCC. We previously demonstrated that the tumor-suppressive microRNA (miRNA) *miR-634* functions as a negative modulator of the cytoprotective cancer cell survival processes and is a useful anticancer therapeutic agent. In the present study, we found that topical application of an ointment containing *miR-634* inhibited *in vivo* tumor growth without toxicity in a cSCC xenograft mouse model and a 7,12-dimethylbenz[*a*]anthracene (DMBA)/12-*O*-tetradecanoylphorbol-13-acetate (TPA)-induced papilloma mouse model. Functional validation revealed that *miR-634* overexpression reduced glutaminolysis by directly targeting *ASCT2*, a glutamine transporter. Furthermore, overexpression of *miR-634* synergistically enhanced TKI-induced cytotoxicity by triggering severe energetic stress *in vitro* and *in vivo*. Thus, we propose that topical treatment with *miR-634* ointment is a useful strategy for improving for EGFR TKI-based therapy for cSCC.

INTRODUCTION

Cutaneous squamous cell carcinoma (cSCC) is the second most common form of human cancer, and its annual incidence is increasing.[1] Outcomes in patients with cSCC are excellent in terms of survival; more than 90% of cSCCs are cured by initial treatment, such as surgical excision, cryosurgery, and radiotherapy.[1–4] However, surgery may not be the best option for patients with numerous or extensive lesions, elderly patients, or patients who are concerned about cosmetic outcomes.[4,5] While topical medications, including the topical chemotherapeutic agent 5-fluorouracil (5-FU), have been used as nonsurgical treatments for cSCC, the long treatment duration and severe side effects limit patient compliance and, consequently, therapeutic efficacy.[5–9] Thus, it is necessary to develop more effective and safer topical treatments for patients with cSCC.

Epidermal growth factor receptor (EGFR) signaling is one of the most intensely studied determinants of epithelial cell proliferation and is persistently activated in various human cancers, including cSCC.[10,11] EGFR overexpression is observed in 70% of cSCCs and is associated with poor outcome in patients with cSCC.[10] EGFR inhibitors, including monoclonal antibodies (e.g., cetuximab and panitumumab) and tyrosine kinase inhibitors (TKIs; e.g., gefitinib and erlotinib), have been evaluated in clinical trials for the treatment of advanced cSCC but have shown only partial clinical benefit.[3,12–15] Notably, a phase II study of gefitinib, which was initially studied in chemotherapy-refractory non-small-cell lung cancer (NSCLC), recently demonstrated modest activity in advanced cSCC, with an overall response rate of 16%.[14] TKI-induced cytotoxicity is attenuated by various cytoprotective cell survival processes, including anti-apoptosis signaling, antioxidant scavenging, and autophagy.[16–19] Furthermore, glutaminolysis, a metabolic process involving glutamine utilization that produces ATP as an energy source and glutathione (GSH) as a reactive oxygen species (ROS) scavenger, is also involved in attenuating TKI-induced cytotoxicity.[20,21] Therefore, negatively modulating these cytoprotective processes may be a reasonable strategy for improving the efficacy of EGFR TKIs in cSCC.

MicroRNAs (miRNAs) are endogenous, small noncoding RNAs that can negatively regulate gene expression by interfering with the translation and/or stability of multiple target transcripts through direct binding to the 3′ untranslated region (UTR), thereby modulating numerous biological processes.[22,23] Given that tumor-suppressive miRNAs (TS-miRNAs) have the potential to simultaneously regulate multiple targets in cancer-related cascades, the therapeutic use of TS-miRNAs could represent a novel anticancer strategy.[24,25] We previously identified multiple TS-miRNAs that inhibit human cancer cell growth, cell invasion, and chemoresistance.[26–34] Notably,

Received 2 August 2020; accepted 17 October 2020;
https://doi.org/10.1016/j.omto.2020.10.009.

Correspondence: Johji Inazawa, Department of Molecular Cytogenetics, Medical Research Institute, Tokyo Medical and Dental University, 1-5-45 Yushima, Bunkyo-ku, Tokyo 113-8510, Japan.
E-mail: johinaz.cgen@mri.tmd.ac.jp

 © 2020 The Author(s).
This is an open access article under the CC BY license (http://creativecommons.org/licenses/by/4.0/).

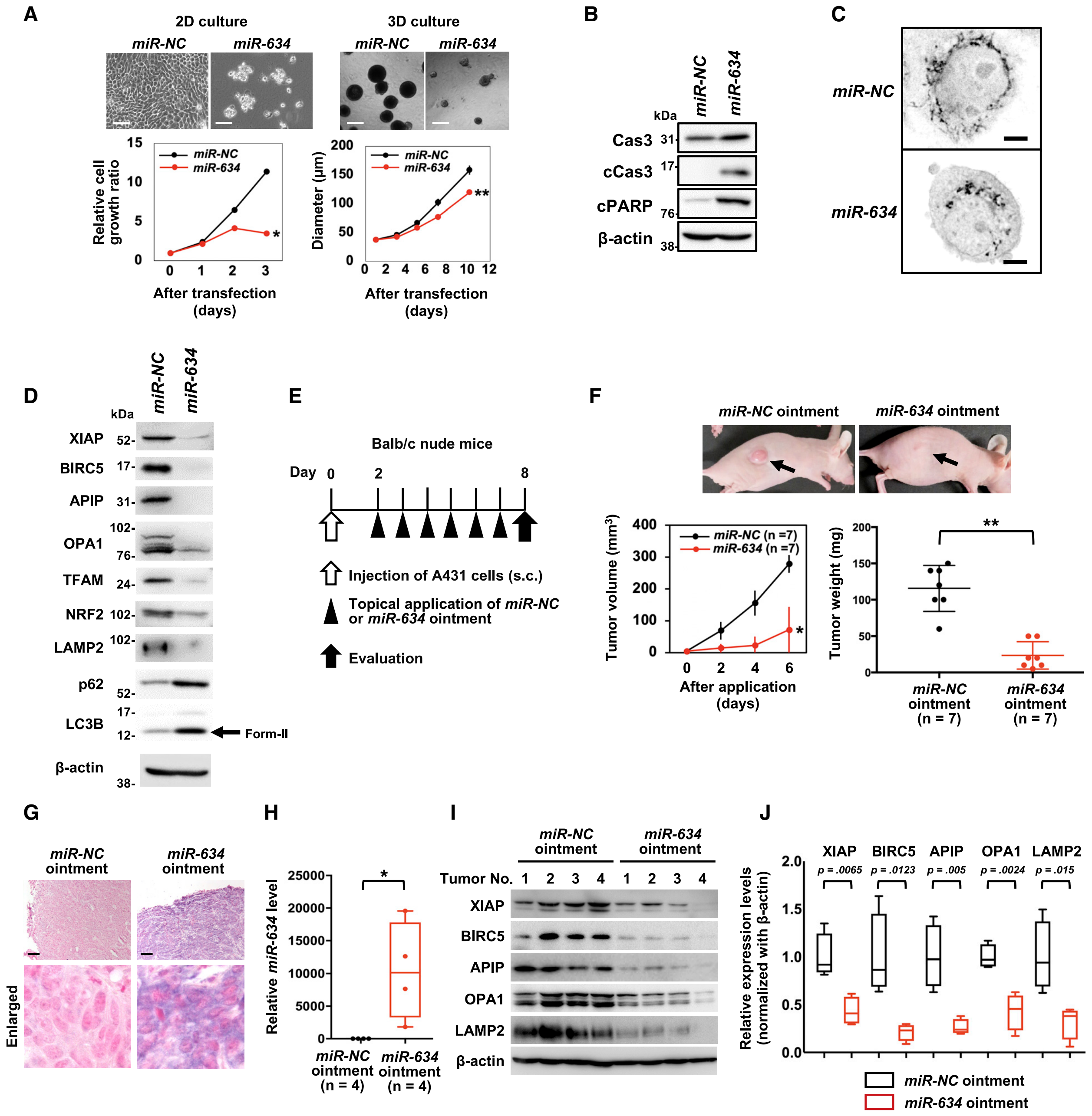

Figure 1. Therapeutic Potential of *miR-634* Ointment for cSCC

(A) Cell growth assay in 2D or 3D *in vitro*. Scale bars, 2D, 50 µm; 3D, 100 µm. Bars indicate the SD. The error bars are not visible because they are too small. Data are presented as mean ± SD. p values were calculated using the two-sided Student's t test (*p = 0.0001, **p = 0.002). (B) Western blotting analysis of resected tumors. Cell lysates were separated by SDS-PAGE and immunoreacted with the indicated antibodies. (C) Representative images of mitochondrial staining. Images are represented in the grayscale. Scale bars, 2 µm. (D) Western blotting analysis of resected tumors. Cell lysates were separated by SDS-PAGE and immunoreacted with the indicated antibodies. The arrow indicates the band corresponding to LC3B-II, an autophagosome marker. (E) Experimental schedule for therapeutic applications. (F) *In vivo* tumor growth assay. Upper panel: representative images of subcutaneous tumors at day 6 after initial treatment. Lower left panel: tumor volume in mice treated with *miR-NC* ointment (n = 7) or *miR-634* ointment (n = 7). Errors bars indicate the SD. Lower right panel: scatterplot of tumor weight in mice treated with *miR-NC* ointment or *miR-634* ointment. Data are presented as mean ± SD. p values were calculated using the two-sided Student's t test (*p = 0.0017, **p < 0.0001). (G) Representative images of ISH in tumors from mice treated with *miR-NC* ointment or *miR-634* ointment. The *miR-634*-specific probe appears purple in the cytoplasm, and the nucleus was counterstained with nuclear fast red. Scale bars, 100 µm. (H) qRT-PCR analysis of *miR-634* expression in resected tumors. *miR-634* expression levels in tumors from mice treated with *miR-NC* ointment (n = 4) or *miR-634* ointment (n = 4) were measured by qRT-PCR, and the results are shown in the boxplot. P values were calculated using the two-sided Student's t test (*p = 0.0326). (I)

(legend continued on next page)

miR-634 overexpression effectively induced apoptosis in various cancer cells by directly and concurrently targeting multiple genes associated with cytoprotective processes, including those involved in mitochondrial homeostasis (*OPA1* and *TFAM*), anti-apoptosis signaling (*APIP*, *XIAP*, and *BIRC5*), antioxidant scavenging (*NRF2*), and autophagy and lysosomal degradation (*LAMP2*).[29] Furthermore, the overexpression of *miR-634* markedly enhanced chemotherapy-induced cytotoxicity in esophageal squamous cell carcinoma (ESCC) cells.[29] Additionally, the local injection of synthetic double-strand (ds) *miR-634* mimic into ESCC xenograft tumors was shown to be therapeutically effective.[29] Recently, we demonstrated that the intravenous administration of lipid nanoparticles (LNPs) harboring *miR-634* effectively delivered *miR-634* into tumor cells and significantly suppressed *in vivo* tumor growth in a pancreatic xenograft mouse model.[35] Thus, our accumulated findings strongly suggest that *miR-634* functions as a negative modulator of cytoprotective processes and that the ds-*miR-634* mimic is a useful agent for miRNA-based cancer therapeutics. In the present study, we sought to formulate an ointment incorporating ds-*miR-634* mimics (*miR-634* ointment) and evaluate its therapeutic potential in a human cSCC xenograft mouse model and a 7,12-dimethylbenz[*a*]anthracene (DMBA)/12-*O*-tetradecanoylphorbol-13-acetate (TPA)-induced papilloma mouse model. We further aimed to verify the ability of *miR-634* ointment to improve the efficacy of EGFR TKIs for cSCC.

RESULTS

Therapeutic Potential of *miR-634* Ointment in cSCC

We first found that *miR-634* overexpression effectively inhibited the growth of A431 cells, a well-known cSCC cell line, under both 2D and 3D (anchorage-independent) culture conditions (Figure 1A). Apoptotic features, including marked increases in cleaved caspase-3 and PARP levels and mitochondrial injury, as indicated by fragmented morphology, were observed in *miR-634*-overexpressing cells compared with *miR*-negative control (*NC*)-overexpressing cells (Figures 1B and 1C). As we have previously shown that the sensitivity to *miR-634* was very low in normal fibroblasts, *miR-634* overexpression markedly induced apoptosis in A431, whereas not in three normal fibroblasts (WI-38, IMR-90, and TIG-1 cells) (Figure S1).[29,35] The expression levels of *miR-634* target genes, such as XIAP, BIRC5, APIP, OPA1, TFAM, NRF2, and LAMP2, were decreased following *miR-634* overexpression (Figure 1D). In addition, the levels of LC3B-II, an autophagosome marker, and p62, a substrate of autophagic degradation, were increased in *miR-634*-expressing cells, suggesting that *miR-634* impairs autophagy (Figure 1D).[36,37] Thus, *miR-634* effectively triggered the activation of the mitochondrial apoptosis pathway in A431 cells, as previously shown in other cancer cell lines,[29,35] suggesting that ds-*miR-634* mimic is a useful miRNA therapeutic in cSCC.

Topical treatment is therapeutically essential for patients with cSCC who are not candidates for, or who refuse, surgical treatment.[4,5] The ionic liquid transdermal system (ILTS) is used to enhance the transdermal permeability of nucleotides, such as oligonucleotides and small interfering RNA (siRNA), through hydrophobic skin tissue, and it achieves efficient delivery into skin cells.[38,39] Hence, we formulated an ointment incorporating ds-*miR-634* mimics (*miR-634* ointment) by using the ILTS. We then evaluated the therapeutic potential, including antitumor efficacy and safety, of this ointment in an A431 xenograft mouse model. We found that daily topical application of *miR-634* ointment onto the subcutaneous tumor effectively inhibited *in vivo* tumor growth (Figures 1E and 1F). *In situ* hybridization (ISH) and qRT-PCR analyses of tumors resected 6 days after treatment initiation showed that *miR-634* was efficiently delivered into tumor cells (Figures 1G and 1H). Furthermore, when we topically applied an ointment incorporating synthesized Cy3-labeled *miR-634* onto the subcutaneous tumor, a direct immunofluorescence analysis revealed the efficient delivery of Cy3-*miR-634* into tumor cells and skin cells (Figure S2). Importantly, the expression of *miR-634* target genes was downregulated in tumors treated with *miR-634* ointment compared with those treated with *miR-NC* ointment, as shown by western blot and immunohistochemistry (IHC) analyses (Figures 1I and 1J; Figure S3). Additionally, in the DMBA/TPA-induced papilloma model, topical application of *miR-634* ointment inhibited tumor growth subsequent to the efficient delivery of *miR-634* into papilloma tumor cells (Figure S4). These results suggest that the topical application of *miR-634* ointment is an effective therapeutic strategy for cSCC.

Next, we evaluated the safety of topical treatment with *miR-634* ointment in mice. The expression of *miR-634* was slightly increased in the internal organs, including the kidneys, liver, and lungs, of xenograft mice (n = 4) treated with *miR-634* ointment after 6 days of treatment compared with mice (n = 4) treated with *miR-NC* ointment, due to the systemic delivery of exogenous *miR-634* through the topical treatment (Figure 2A). However, there were no significant differences in the levels of aspartate aminotransferase (AST) and alanine aminotransferase (ALT), markers of liver injury, in the plasma of mice treated with *miR-634* ointment or *miR-NC* ointment or control mice (no treatment) (Figure 2B). To evaluate immune-mediated toxicity, an adverse event associated with nucleic acid therapeutics, we topically applied either *miR-NC* ointment or *miR-634* ointment on the skin of BALB/c mice with a normal immune system 7 days after depilation (Figure 2C). After 6 days of treatment, *miR-634* expression was increased in skin treated with *miR-634* ointment compared with skin treated with *miR-NC* ointment or control (no treatment) skin; however, there were no pathological changes, such as immune cell infiltration into skin tissue (Figures 2D and 2E). Additionally, the serum levels of proinflammatory cytokines, including tumor necrosis factor (TNF)-α, interferon (IFN)-γ, and interleukin (IL)-6, showed no significant differences between mice treated with *miR-634* ointment and those treated with *miR-NC* ointment or control mice (no treatment) (Figure 2F). Taken together, these findings suggest that topical treatment with *miR-634* ointment is a therapeutically effective and safe option for cSCC.

Western blotting analysis of resected tumors. Cell lysates were separated by SDS-PAGE and immunoreacted with the indicated antibodies. (J) Relative expression levels of target genes. The expression levels of target genes in tumors treated with *miR-634* ointment relative to those treated with *miR-NC* ointment are presented as boxplots. P values were calculated using the two-sided Student's t test.

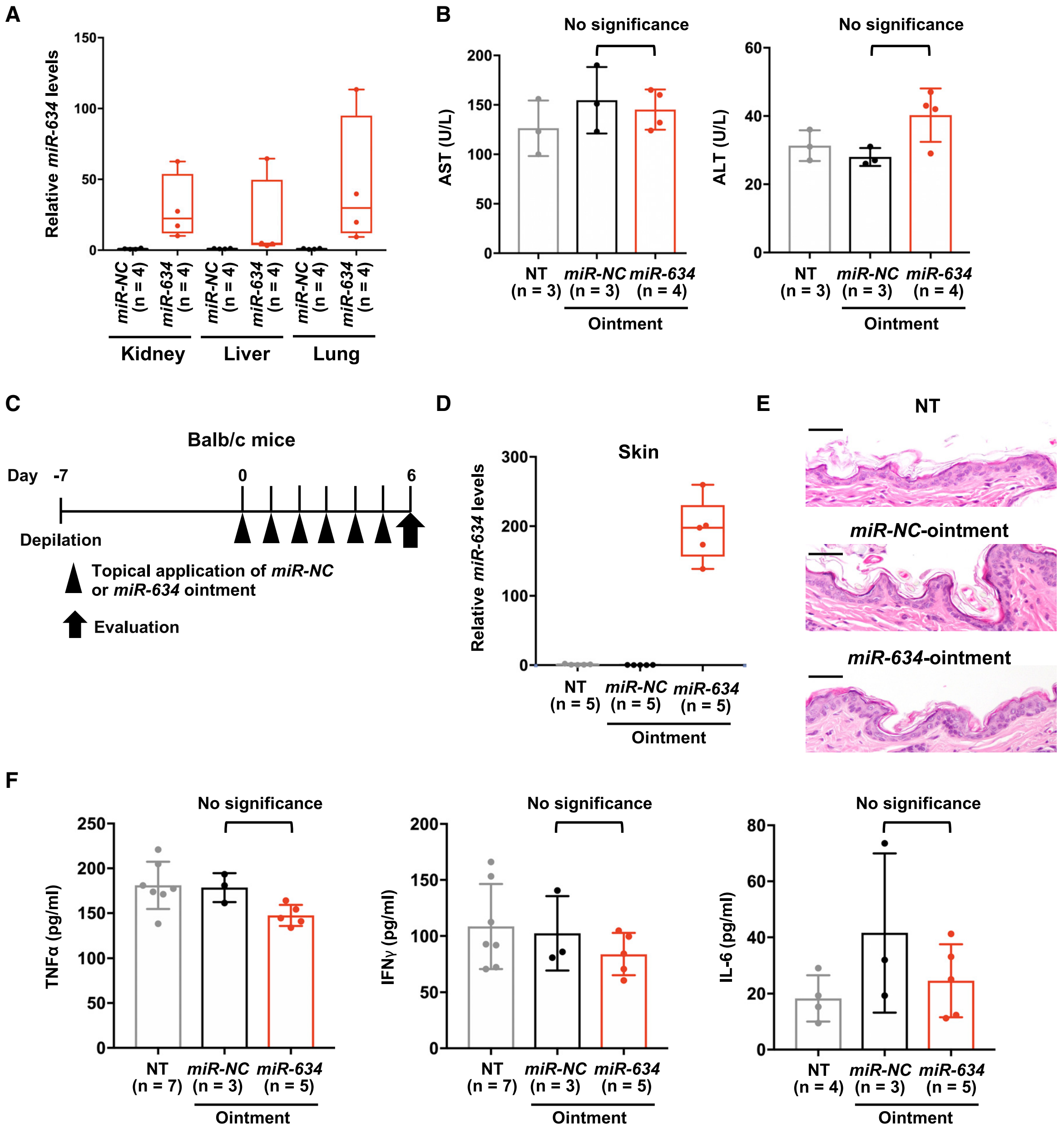

Figure 2. Safety of the Topical Application of *miR-634* Ointment in Mice

(A) qRT-PCR expression analysis of *miR-634* in the kidneys, liver, and lungs. *miR-634* expression levels in normal tissues from mice treated with miR-NC ointment (n = 4) or *miR-634* ointment (n = 4) were measured by qRT-PCR, and the results are presented in the boxplot. (B) Plasma AST (left) and ALT (right) levels in mice treated with *miR-NC* ointment (n = 3) or *miR-634* ointment (n = 4) at 6 days after initial treatment and in control mice (no treatment [NT]) (n = 3). Error bars indicate the SD. Data are presented as mean ± SD. p values were calculated using the one-way ANOVA (p = 0.893 for AST, p = 0.065 for ALT). (C) Experimental schedule for the application of *miR-634* ointment in BALB/c mice. (D) *miR-634* expression analysis in skin tissues by qRT-PCR. *miR-634* levels in skin tissues from mice treated with *miR-NC* ointment (n = 5) or *miR-634* ointment (n = 5) and from control mice (NT) (n = 5) were measured by qRT-PCR, and the results are presented in the boxplot. (E) H&E staining of skin tissues from mice treated with *miR-NC* ointment or *miR-634* ointment and from control mice (NT). Scale bars, 50 μm. (F) Serum TNF-α, IFN-γ, and IL-6 levels in mice treated with *miR-NC* ointment or *miR-634* ointment at 6 days after initial application and in in control mice (NT). Error bars indicate the SD. Data are presented as mean ± SD. p values were calculated using the one-way ANOVA (p = 0.145 for TNF-α, p = 0.713 for IFN-γ, and p = 0.381 for IL-6).

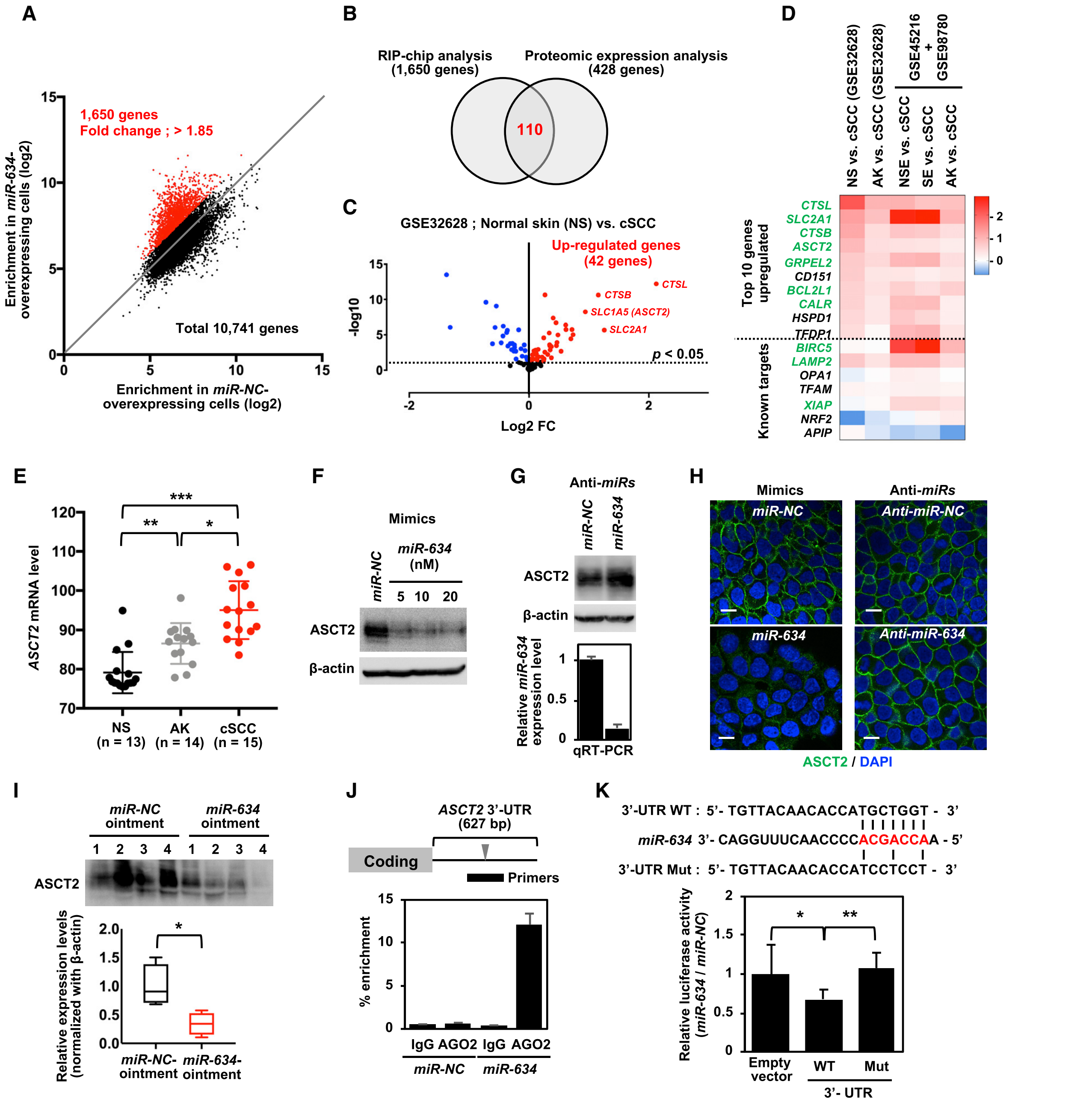

Figure 3. Identification of *ASCT2/SLC1A5* as a *miR-634* Target Gene

(A) Correlation plot of enrichment (RIP/input) of 10,741 genes between *miR-NC*-overexpressing cells and *miR-634*-overexpressing cells in the RIP-chip analysis. A total of 1,650 genes were extracted as enriched in the RIP following *miR-634* overexpression (red). (B) Proteomic expression analysis identified 428 genes that were downregulated at the protein level in *miR-634*-overexpressing cells compared with *miR-NC*-overexpressing cells (fold change [FC] < 1.0). Finally, 110 genes identified by both analyses were defined as directly targeted by *miR-634* in A431 cells. (C) Volcano plot of $\log_2$ ratio versus p value of differentially expressed genes between primary cSCC samples (n = 15) and normal skin (NS; n = 13) on an expression array dataset (GEO: GSE32628). Significantly upregulated or downregulated genes were indicated as red or blue dots, respectively. The dotted line indicates an adjusted p value threshold of 0.05. (D) Heatmap of $\log_2$ FCs of cSCC versus non-cancerous tissues from GEO: GSE32628 and a combined dataset of GEO: GSE98780 and GSE45216. AK, actinic keratosis; NSE, non-sun-exposed NS; SE, sun-exposed NS. Significantly upregulated genes in four out of five comparisons are highlighted in green. Within the heatmap, red color indicates the upregulation in cSCC compared to non-cancerous tissues, and the blue color indicates the downregulation in cSCC. (E) Expression level of *ASCT2* in NS (n = 13, black), AK (n = 14, gray), and cSCC (n = 15, red) from GEO: GSE32628. p values were calculated using the two-sided Student's t test (*p = 0.014 for cSCC versus AK, **p = 0.0006 for NS versus AK, and ***p < 0.0001 for cSCC versus NS). (F) Western blotting analysis of ASCT2 in *miR-634*-overexpressing cells. After 3 days of transfection, cell lysates were prepared, separated by SDS-PAGE, and immunoreacted with the indicated antibodies.

(legend continued on next page)

Identification of the *ASCT2/SLC1A5* Gene as a *miR-634* Target Gene

To further clarify the tumor-suppressive function of *miR-634* and thus support the clinical use of *miR-634* ointment, we identified genes directly targeted by *miR-634* in A431 cells. The RNA-induced silencing complex (RISC), a multiprotein complex comprising a ribonucleoprotein (RNP) such as Argonaute 2 (AGO2), can direct the interaction of miRNA with target mRNA.[40] Hence, we first identified transcripts enriched in RNP immunoprecipitation (RIP) assays using an antibody against AGO2 in cells overexpressing *miR-634* by microarray analysis (RIP-chip analysis).[41] The enrichment indicated by RIP mRNA levels relative to total mRNA levels (input) of 10,741 genes was compared between *miR-NC*-overexpressing cells and *miR-634*-overexpressing cells. We confirmed that known *miR-634* target genes were robustly enriched by RIP in *miR-634*-overexpressing cells compared with *miR-NC*-overexpressing cells (fold change of enrichment: *LAMP2*, 2.0; *TFAM*, 4.66; *APIP*, 8.7; *BIRC5*, 3.27; and *XIAP*, 1.85), suggesting that this analysis is accurate. With these assays, we extracted 1,650 genes that were enriched by RIP in cells overexpressing *miR-634*, with a greater than 1.85-fold change detected in *XIAP* (Figure 3A). Alternatively, iTRAQ-based proteomic expression analysis identified 428 genes that were downregulated at the protein level in *miR-634*-overexpressing cells compared with *miR-NC*-overexpressing cells (fold change < 1.0) (Table S1). Finally, 110 genes identified by both analyses were considered directly targeted by *miR-634* in A431 cells (Figure 3B; Table S2). These genes were significantly associated with mitochondrial organization, protein folding, and amino acid transport in the Gene Ontology (GO) enrichment analysis (Table S3). Furthermore, we found that mRNA expression was significantly upregulated in 42 of 110 genes in primary cSCC samples compared with normal skin (NS) samples on a Gene Expression Omnibus (GEO) expression array dataset (GEO: GSE32628) (Figure 3C). The top 10 genes among them, together with known *miR-634* target genes,[29] were also upregulated in primary cSCCs compared to actinic keratosis (AK), a premalignant precursor for cSCC, in GEO: GSE32628 and NS in other datasets of GEO: GSE98780 and GSE45216 (Figure 3D), suggesting the possible implementation of a functional prediction method for the anti-tumor effect of *miR-634* in patients with cSCCs by using expression profiles of *miR-634* target genes in those tumors. Among these genes, the glutamine transporter *SLC1A5* (also known as *ASCT2*) was verified as a *miR-634* target gene in A431 cells; ASCT2-dependent glutamine uptake and subsequent glutaminolysis metabolism, which involves glutamine utilization, are essential for cancer cell survival and proliferation.[42] In addition, *ASCT2* expression was gradually increased in AK and cSCC compared with NS on an expression array dataset (GEO: GSE32628), suggesting that this gene might be involved in the malignant progression of cSCC (Figure 3E). Overexpression of *miR-634* downregulated the expression of ASCT2, and, conversely, *miR-634* knockdown by transfection with an anti-*miR-634* inhibitor upregulated ASCT2 expression (Figures 3F and 3G). Similarly, immunofluorescence analysis revealed that *miR-634* can negatively regulate the amount of ASCT2 protein localized on the A431 cell membrane (Figure 3H). The expression of ASCT2 was downregulated in tumors treated with *miR-634* ointment compared with those treated with *miR-NC* ointment, as shown by western blot analysis (Figure 3I). Furthermore, we identified putative *miR-634* binding sites within the 3′ UTR of the *ASCT2* gene and confirmed by RIP-PCR analysis that *ASCT2* mRNA was enriched by RIP with the anti-AGO2 antibody but not with normal immunoglobulin G (IgG) antibody (negative control) in cells overexpressing *miR-634* (Figure 3J). The luciferase activity of the wild-type (WT) 3′ UTR vector was significantly reduced compared to that of the empty vector, and this reduction was completely restored by mutation of a putative binding site (Figure 3K). These findings suggest that *miR-634* can downregulate *ASCT2* expression by directly binding its 3′ UTR. In addition, the uptake of $^{15}N_2$-labeled glutamine was somewhat reduced following *miR-634* overexpression compared with *miR-NC* expression, as well as following the siRNA-mediated knockdown of *ASCT2* (Figure S5). Thus, these results suggest that *miR-634* can suppress glutaminolysis by directly targeting *ASCT2* in A431 cells.

Augmentation of TKI-Induced Cytotoxicity by *miR-634*

EGFR TKIs (gefitinib and erlotinib) have been evaluated in clinical trials for the treatment of advanced cSCC but have shown only partial clinical benefit.[3,12–15] Various cytoprotective processes, including glutaminolysis, are involved in attenuating TKI-induced cytotoxicity.[16–19] Hence, we examined whether overexpression of *miR-634* enhances TKI-induced cytotoxicity. When A431 cells were transfected with increasing doses (1.0–10 nM) of *miR-634* and simultaneously treated with gefitinib (1–30 μM), capase-3/7 activity gradually increased in a dose-dependent manner compared with cells treated with gefitinib only (Figure 4A). The cell survival rate was reduced by treatment with combinations of gefitinib and *miR-634* at various doses, and analysis of the combination index (CI) indicated that *miR-634* significantly synergized with gefitinib in A431 cells (Figures 4B and 4C).

(G) Western blotting analysis of ASCT2 and expression analysis of *miR-634* in *miR-634*-inhibited cells. A431 cells were transfected with 100 nM anti-*miR-NC* or anti-*miR-634*. Upper panel: after 3 days of transfection, cell lysates were prepared, separated by SDS-PAGE, and immunoreacted with the indicated antibodies. Lower panel: qRT-PCR analysis of *miR-634* expression. Error bars indicate the SD. Data are presented as mean ± SD. (H) Immunofluorescence analysis of ASCT2. After 3 days of transfection, cells were fixed, blocked, and incubated with anti-ASCT2 antibodies. Images were obtained by confocal fluorescence microscopy. Scale bars, 10 μm. (I) Western blotting analysis of resected tumors. The cell lysates described in Figure 1I were separated by SDS-PAGE and immunoreacted with anti-ASCT2 antibodies. The expression levels of target genes in tumors treated with *miR-634* ointment relative to those treated with *miR-NC* ointment are indicated as boxplots. p values were calculated using the two-sided Student's t test (*p = 0.019). (J) RIP-PCR analysis of the *ASCT2* gene. Upper panel: a putative *miR-634* binding site within the 3′ UTR of the *ASCT2* gene. Lower panel: enrichment relative to IgG immunoprecipitation in *miR-NC*-overexpressing cells. Data are presented as mean ± SD. (K) Luciferase assays using reporter plasmids. The luciferase activity in *miR-634*-transfected cells relative to that in *miR-NC*-transfected cells is indicated on the vertical axis. Error bars indicate the SD. Data are presented as mean ± SD. p values were calculated using one-way ANOVA (*p = 0.024 to empty vector, **p = 0.0046 to Mut vector).

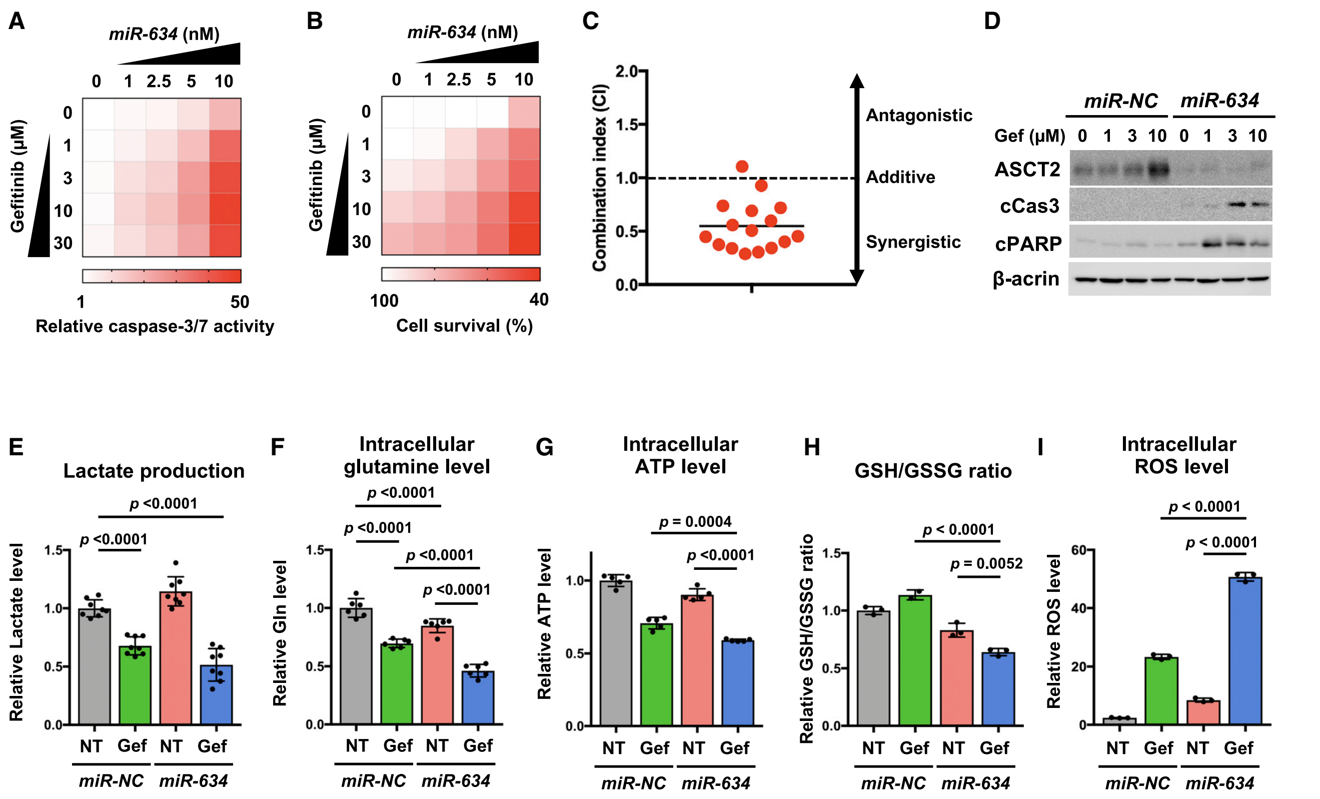

Figure 4. Augmentation of Gefitinib-Induced Cytotoxicity by *miR-634*

(A) Caspase-3/7 activity assay. After 24 h of gefitinib treatment, caspase-3/7 activity was measured and normalized to the cell survival rate in each combined treatment group, and the results are reported as the relative rate compared with non-transfected and non-treated cells. (B) Cell survival assay. Cells were treated as described in (A). The results are reported as the relative rate compared with non-transfected and non-treated cells. (C) Combination index for A431 cells treated with gefitinib and *miR-634*. (D) Western blotting analysis of ASCT2 and apoptosis markers. Cell lysates were separated by SDS-PAGE and immunoreacted with the indicated antibodies. (E–I) Metabolite analysis of A431 cells treated with gefitinib (Gef) and/or *miR-634*. A431 cells were transfected with 10 nM *miR-NC* or *miR-634* and simultaneously treated with gefitinib (10 μM) for 24 h. Lactate production (E), intracellular glutamine levels (F), intracellular ATP levels (G), the GSH/GSSG ratio (H), and intracellular ROS levels (I) were measured and normalized to the cell survival rate. Error bars indicate the SD. Data are presented as mean ± SD. p values were calculated using two-way ANOVA.

Furthermore, ASCT2 expression was upregulated following treatment with gefitinib and downregulated by overexpression of *miR-634*, accompanied by increases in cleaved caspase-3 and cleaved PARP levels, as shown by western blotting (Figure 4D). This synergistic effect was also observed upon combined treatment with *miR-634* and erlotinib, another EGFR TKI (Figure S6). Thus, these findings suggest that the overexpression of *miR-634* synergistically enhances TKI-induced cytotoxicity in A431 cells.

To further understand the mechanism of synergism between *miR-634* and EGFR TKIs, we next assessed the changes in metabolite levels. TKIs potently suppress glycolysis,[43,44] the metabolic pathway that produces ATP and various metabolites from glucose. Indeed, we confirmed decreased levels of lactate, a final product of glycolysis, in gefitinib-treated A431 cells (*miR-NC* + gefitinib and *miR-634* + gefitinib) (Figure 4E). Furthermore, we found that intracellular glutamine levels were moderately decreased by treatment with *miR-NC* + gefitinib or *miR-634* + nontreatment (NT), and this decrease was markedly enhanced by combined treatment of A431 cells with *miR-634* + gefitinib (Figure 4F). Glutaminolysis, the metabolic pathway for intracellular glutamine utilization, produces ATP as an energy source by fueling the mitochondrial tricarboxylic acid (TCA) cycle and GSH as a ROS scavenger. Corresponding with the decreased intracellular glutamine pool,[45] the production of ATP and GSH, as indicated by a decrease in the ratio with oxidized GSH (GSSG), was markedly reduced by combined treatment with *miR-634* + gefitinib compared with *miR-NC* + gefitinib or *miR-634* + NT (Figures 4G and 4H). Incidentally, ROS accumulation was most apparent in cells treated with *miR-634* + gefitinib (Figure 4I). Thus, these findings suggest that overexpression of *miR-634* can synergistically enhance TKI-induced cytotoxicity by triggering severe energetic stress, accompanied by a marked decrease in the intracellular glutamine pool following the reduction in ATP and GSH and marked ROS accumulation. This energetic stress was also observed in cells treated with both *miR-634* and erlotinib (Figure S6). Additionally, we showed that siRNA-mediated *ASCT2* knockdown enhanced gefitinib-induced cytotoxicity, accompanied by a reduction in ATP production, suggesting that the *miR-634*-mediated inhibition of *ASCT2*

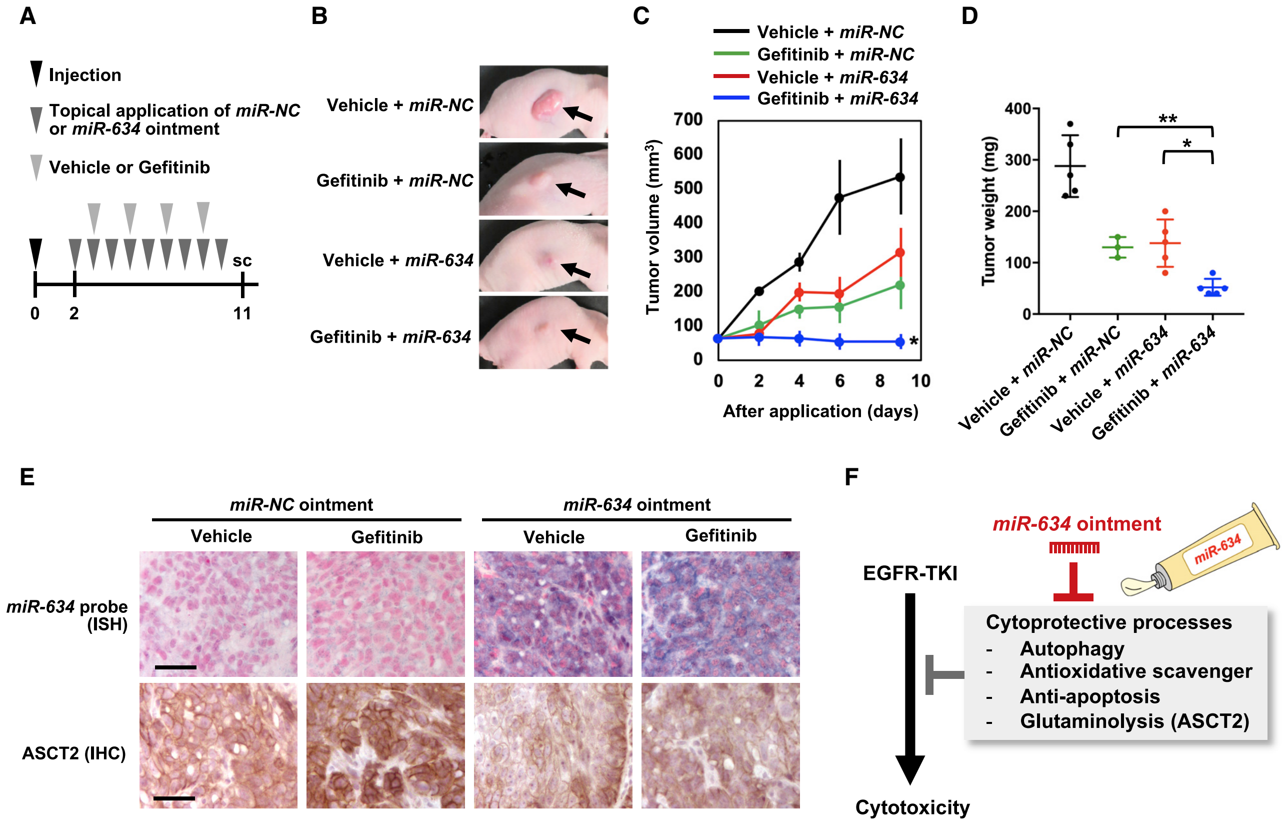

Figure 5. Improvement in the *In Vivo* Efficacy of Gefitinib by *miR-634* Ointment

(A) Experimental schedule for the application of *miR-634* ointment and treatment with gefitinib. Mice were treated orally with vehicle (0.5% methylcellulose) or gefitinib (10 mg/kg) on days 3, 5, 7, and 9 after cell injection. At 9 days after the initial application, the mice were sacrificed, and the tumors were evaluated. (B) Representative images of subcutaneous tumors after 9 days of treatment. (C) Tumor volume in mice treated with vehicle + *miR-NC* ointment (n = 5), gefitinib + *miR-NC* ointment (n = 3), vehicle + *miR-634* ointment (n = 5), or gefitinib + *miR-634* ointment (n = 5). Error bars indicate the SD. Data are presented as mean ± SD. p values for the treatment with *miR-634* ointment + gefitinib were calculated using two-way ANOVA (p = 0.043 for *miR-NC* ointment + gefitinib and p = 0.0005 for *miR-634* ointment + vehicle). (D) The tumor weight in mice treated with *miR-NC* ointment or *miR-634* ointment is presented in the scatterplot. p values were calculated using two-way ANOVA (*p = 0.0092, **p = 0.043). (E) ISH analysis of *miR-634* and IHC analysis of ASCT2 in resected tumors. The *miR-634*-specific probe appears purple in the cytoplasm, and the nucleus was counterstained with nuclear fast red. Scale bars, 50 μm. (F) Therapeutic potential of *miR-634* ointment to improve the efficacy of EGFR inhibitors.

partially contributes to the synergism with energetic stress (Figure S7).

Improvement in Gefitinib Efficacy by Topical Treatment with *miR-634* Ointment *In Vivo*

Finally, we examined whether topical application of *miR-634* ointment improves the therapeutic effect of gefitinib *in vivo*. *miR-NC* ointment or *miR-634* ointment was topically applied to the subcutaneous tumors daily for 9 days (days 2–11 after cell injection), and mice were treated orally with vehicle or gefitinib on days 3, 5, 7, and 9 after cell injection (Figure 5A). Tumor growth was effectively inhibited in mice treated with gefitinib + *miR-634* ointment compared with mice treated with gefitinib + *miR-NC* ointment or vehicle + *miR-634* ointment (Figures 5B–5D). In addition, the delivery of *miR-634* into tumor cells and the downregulation of ASCT2 protein levels were confirmed in tumors treated with *miR-634* ointment (Figure 5E).

Various cytoprotective cell survival processes, including anti-apoptosis signaling, antioxidant scavenging, autophagy, and glutaminolysis, are involved in attenuating TKI-induced cytotoxicity.[16–21] Our current and previous studies demonstrated that *miR-634* can directly and concurrently target multiple genes associated with these cytoprotective processes, including *ASCT2* in glutaminolysis. Therefore, we propose that topical treatment with *miR-634* ointment is a reasonable strategy for maximizing the efficacy of TKI-based therapy in cSCC through the concurrent modulation of multiple cytoprotective processes (Figure 5F).

DISCUSSION

To develop a novel therapeutic option for patients with cSCC who are not candidates for, or who refuse, surgical treatment, we formulated an ointment incorporating ds-*miR-634* mimics as an anticancer agent and showed that topical application of this ointment onto subcutaneous tumors inhibited *in vivo* tumor growth without toxicity in a

human cSCC xenograft mouse model and a DMBA/TPA-induced papilloma mouse model. Thus, our findings in the current study strongly suggest the therapeutic potential of *miR-634* ointment for cSCC.

Considering the potential clinical use of *miR-634* ointment, it is important to define the toxicity of synthesized ds-*miR-634* mimics in the body. Similar to other nucleotide therapeutics involving oligonucleotides and siRNA, miRNA therapeutics carry the risk of unintended toxicity through two mechanisms: hybridization-independent and hybridization-dependent effects.[46–48] Immune-mediated toxicity is a typical adverse event caused by immune activation through the recognition of artificial nucleotides in immune cells that occurs through a hybridization-independent mechanism.[49] Indeed, a phase I study of the systemic administration of MRX34, a liposomal *miR-34a* mimic, in patients with advanced solid tumors showed adverse immune responses.[50] In the current study, we did not observe any features associated with immune-mediated toxicity, such as immune cell infiltration of skin tissue and proinflammatory cytokine production, in mice topically treated with *miR-634* ointment. Notably, the delivery of *miR-634* into the internal organs, including the kidneys, liver, and lungs, of mice treated with *miR-634* ointment was quite limited. Thus, topical treatment is potentially less toxic to patients than systemic treatment but also allows for higher local *miR-634* levels at the tumor site. However, the safety for *miR-634* ointment, as well as the optimization of the therapeutic schedule and dosage, should be evaluated further in a large cohort of mice and multiple experimental models, including the DMPA/TPA-induced skin papilloma mouse model, for the clinical application of this therapeutic option.

Nevertheless, when *miR-634* is delivered into NS cells following the topical application of *miR-634* ointment, there is the possibility of hybridization-dependent toxicity due to the direct downregulation of multiple target genes in healthy cells. Our results showed no changes in any pathological structures or findings in the NS of mice topically treated with *miR-634* ointment. In addition, our previous study demonstrated that *miR-634*-induced cell death does not occur in normal cells, such as human fibroblasts, suggesting that the effect of *miR-634* may be specific to cancer cells.[29] Furthermore, in the current study, we identified 110 genes that were directly targeted by *miR-634*. Hence, understanding the expression profiles of these target genes in various normal tissues, including skin, would help predict the hybridization-dependent toxicity of *miR-634* ointment clinically applied to humans.

Cancer cells utilize various metabolic pathways, including glycolysis and glutaminolysis, to meet the bioenergetic and biosynthetic demands for survival.[51] EGFR TKIs potently suppress glycolysis and pharmacological inhibition of glutaminase, which facilitates the conversion of glutamine to glutamate and augments erlotinib-induced cytotoxicity by triggering energetic stress, suggesting that dual inhibition of glycolysis and glutaminolysis is a promising therapeutic anticancer strategy.[20,21,43,44] Consistent with this idea, we found that the suppression of glutaminolysis through *miR-634*-mediated inhibition of *ASCT2* augmented TKI-induced cytotoxicity by triggering severe energetic stress, accompanied by a decrease in intracellular glutamine levels and a subsequent reduction in ATP and GSH and marked accumulation of ROS. Thus, the dual inhibition of glutaminolysis and glycolysis by combined treatment with *miR-634* ointment and a TKI is a reasonable strategy for improving the efficacy of EGFR TKIs in cSCC.

Nonsurgical options for systemic therapy, including chemotherapy, EGFR inhibitors, and anti-PD-1 antibodies, are used to treat cSCC that cannot be cured by surgery or radiotherapy due to the presence of numerous or extensive lesions and/or distant metastasis.[52] Anti--PD-1 antibodies such as cemiplimab-rwlc appear to achieve higher response rates than do EGFR inhibitors and chemotherapy, and in 2018, cemiplimab-rwlc was approved by the US Food and Drug Administration (FDA) as the first medication for advanced cSCC. Therefore, PD-1 inhibitors are expected to become the new standard of care for patients with locally advanced and metastatic cSCC.[52–55] However, patients not eligible for anti-PD-1 treatment, e.g., organ transplant recipients or patients refractory to anti-PD-1 therapy, may be offered EGFR inhibitors and/or chemotherapy.[52,56] EGFR inhibitors have a more favorable toxicity profile than chemotherapy and hence are more suitable for multimorbid and/or frail, elderly patients.[52] In this context, combined treatment with *miR-634* ointment and EGFR inhibitors may achieve improved response rates and durations compared to EGFR inhibitors alone in locally advanced cSCC.

The ILTS can enhance the transdermal permeability of nucleotides in the hydrophobic field of skin tissue.[57,58] Indeed, topical applications of ILTS-mediated ointments incorporating antisense oligonucleotides or siRNA onto mouse skin have achieved efficient knockdown of target genes in skin cells.[38,39] By using the ILTS, we successfully achieved efficient permeability and delivery of *miR-634* into tumor cells in xenograft mice and DMBA/TPA-induced mouse papilloma cells. In general, RNA molecules are unstable due to nuclease-mediated degradation in the skin, as well as in blood circulation and other bodily fluids.[59] Therefore, chemical modification during the synthesis of ds-*miR-634* mimics can further enhance *miR-634* delivery by increasing resistance to nucleases in the skin.[60] Thus, it is necessary to optimize the *miR-634* ointment formulation at the preclinical stage prior to first-in-human studies for cSCC.

MATERIALS AND METHODS

Cell Culture

A431 cells were obtained from the American Type Culture Collection (ATCC, Manassas, VA, USA) and normal human fibroblasts (WI-38, TIG-1, and IMR-90 cells) were obtained from the JCRB cell bank (Tokyo, Japan). The cultures were maintained in DMEM (Wako, Tokyo, Japan) at 37°C with 5% CO_2. Once resuscitated, the cell lines were authenticated by monitoring cell morphology.

Antibodies and Reagents

Antibodies against the following proteins were used: caspase-3 (#9662), cleaved caspase-3 (#9661), cleaved PARP (#9541), XIAP

(#2042), and BIRC5 (#2808) (Cell Signaling Technology, Danvers, MA, USA); β-actin (A5441), LC3B (L7543), and TFAM (SAB1401383) (Sigma, St. Louis, MO, USA); APIP (ab98153), OPA1 (ab42364), and LAMP2 (ab18529) (Abcam, Cambridge, UK); NRF2 (sc-13032) and p62/SQSTM1 (sc-28359) (Santa Cruz Biotechnology, Dallas, TX, USA); and ASCT2 (Proteintech, Rosemont, IL, USA). Gefitinib and erlotinib were purchased from Tokyo Chemical Industry (Tokyo, Japan) and Wako (Tokyo, Japan), respectively. The stable isotope L-glutamine-$^{15}N_2$ was purchased from Taiyo Nippon Sanso (Tokyo, Japan).

miRNA and siRNA Synthesis and Transfection

The *miRVana miR-634* mimic (4464066) and negative control 1 (*miR-NC*; 4464058), anti-*miR-634* (AM17000), and anti-*miR-NC* (AM17010) were obtained from Thermo Fisher Scientific (Waltham, MA, USA). The siRNA targeting *ASCT2* (pool of four sequences; siGENOME SMARTpool; M-007429-01) and negative control (*siNC*; siGENOME SMARTpool; D-001206-14) were obtained from Dharmacon (Lafayette, CO, USA). Cells were transfected with miRNA or siRNA using Lipofectamine RNAiMAX (Invitrogen, Carlsbad, CA, USA) according to the manufacturer's instructions.

Cell Survival Assay

Cell survival was assessed by crystal violet (CV) staining. The cells were washed in PBS and fixed with 0.1% CV (Wako, Tokyo, Japan) and 4% formaldehyde in PBS for 3 min. The excess CV solution was discarded, and the stained cells were completely air-dried and then lysed in the plates with 2% SDS (Wako) by shaking for 1 h. The optical density (OD) was measured at 560 nm using a microplate reader (Synergy H1; BioTek, VT, USA) The percentage absorbance in each well was determined. The OD values of cells in control wells were arbitrarily set at 100% to determine the percentage of viable cells.

3D Spheroid Culture

Cells were transfected with 10 nM *miR-NC* or *miR-634*. After 5 h of transfection, the cells were suspended in standard medium containing 2% Matrigel and seeded in 96-well plates (3.0×10^3 cells/well) precoated with 75 μL/well of Matrigel (BD Biosciences, San Jose, CA, USA) as a basement membrane by incubation at 37°C for 30 min. All microscopy images were captured using a Nikon Eclipse E400 (Nikon, Tokyo, Japan), and the spheroid size was measured using ImageJ software (National Institutes of Health, MD, USA).

Determination of the Apoptotic Cell Population

Apoptotic cells were stained with the MEBCYTO apoptosis kit (MBL, Nagoya, Japan), and cell population analysis was performed using an Accuri flow cytometer (Becton Dickinson, San Jose, CA, USA). The median fluorescence intensity was calculated using FlowJo software.

Formulation of miRNA ointments

The ILTS (MEDRx, Kagawa, Japan) was used to formulate the ointments incorporating miRNAs. With this approach, ionic liquid is prepared from organic acids and amines. The molecular assembly involves the equilibrium reaction of ionic liquid/acid/amines and hydrogen bond interactions to enhance the transdermal permeability of drugs or nucleotides in the hydrophobic field of skin tissue. The 0.2% ointment incorporating ds-*miR-NC* mimic or ds-*miR-634* mimic was formulated (2 mg of miRNA/mL of ointment), and 10–20 μL of ointment (20–40 μg of miRNA) was topically applied every day onto subcutaneous tumors in A431 xenograft mice, skin in BALB/c mice, or papillomas in FVB/NJcl mice.

In Vivo Tumor Growth Assay

Six-week-old female BALB/c nude mice were purchased from Charles River Laboratories (Yokohama, Japan). A total of 4.0×10^6 A431 cells in 100 μL of PBS were subcutaneously injected into the flanks of the mice. On day 2 after tumor cell inoculation, the topical application of miRNA ointment (*miR-NC* or *miR-634*) to subcutaneous tumors was initiated. Tumor volume was calculated using the following formula: $4/3 \times \pi \times$ (shortest diameter)^2 $\times$ (longest diameter)/2. For animal studies, experiments for the xenograft mouse model or for the DMBA/TPA-induced papilloma mouse model were approved by the Tokyo Medical and Dental University Animal Care and Use Committee or the Committee on the Ethics of Animal Experiments of Nihon University School of Medicine, respectively.

ISH Analysis

The ISH assay was performed on formalin-fixed, paraffin-embedded (FFPE) tissue sections according to the manufacturer's instructions (miRCURY LNA miRNA ISH optimization kit; Exiqon, Vedbaek, Denmark). In brief, the sections were deparaffinized in xylene, rehydrated with a graded ethanol series, and incubated with proteinase K (Exiqon) for 10 min at 37°C. Then, the sections were hybridized with digoxigenin (DIG)-labeled *miR-634* probes (Exiqon) for 1 h at 55°C, washed stringently, incubated with blocking agent for 15 min, and probed with a specific anti-DIG antibody (Sigma) directly conjugated to alkaline phosphatase (AP; Roche, Basel, Switzerland). AP converts the soluble substrates 4-nitro-blue tetrazolium (NBT) and 5-bromo-4-chloro-indolyl phosphate (BCIP) into a dark blue water- and alcohol-insoluble NBT-BCIP precipitate. Finally, the sections were counterstained with nuclear fast red (Vector Laboratories).

IHC and Hematoxylin and Eosin (H&E) Staining

The sections were deparaffinized in xylene and rehydrated using a graded ethanol series (100%, 90%, 80%, 70%, and 50%) in water. After antigen retrieval by boiling in 10 mM citrate buffer (pH 6.0), the sections were treated with 0.3% hydrogen peroxide in methanol to inactivate endogenous peroxidase. Nonspecific binding was blocked by incubation with goat serum in PBS. Next, the slides were incubated overnight at room temperature with rabbit antibodies against BIRC5, XIAP, APIP, and ASCT2. The bound antibody was visualized using diaminobenzidine as the chromogen (Vectastain Elite ABC kit, Vector Laboratories), and the sections were lightly counterstained with hematoxylin (Wako). The sections were also sequentially stained with H&E (Wako).

qRT-PCR

Total RNA was isolated using TRIsure reagent (Nippon Genetics, Tokyo, Japan) according to standard procedures. Single-stranded cDNA

was generated from the total RNA using a PrimeScript II first-strand cDNA synthesis kit (Takara Bio, Shiga, Japan). Quantitative real-time RT-PCR was performed using an ABI Prism 7500 Fast real-time PCR system, TaqMan universal PCR master mix, a TaqMan reverse transcription kit, and TaqMan miRNA assays (*miR-634*; assay ID: 001576; Applied Biosystems, Waltham, MA, USA) according to the manufacturer's instructions. Gene expression values are presented as the ratio (difference in threshold cycle [Ct] values) between *miR-634* and an internal reference, *RNU6B* (for human; assay ID: 001093) or *snoRNA202* (for mouse; assay ID: 001232).

Western Blotting

Whole-cell lysates were separated by SDS-PAGE, and the proteins were transferred to polyvinylidene fluoride (PVDF) membranes (GE Healthcare, Chicago, IL, USA). To detect LAMP2, cell lysates were prepared under nonreducing conditions (without 2-mercaptoethanol [2-ME]). After being blocked with Tris-buffered saline (TBS) containing 0.05% Tween 20 (Sigma) and 5% nonfat dry milk (Becton Dickinson) for 1 h, the membrane was incubated overnight with primary antibodies, washed, and incubated for 3 h with horseradish peroxidase (HRP)-conjugated anti-mouse or anti-rabbit IgG secondary antibody (both at 1/4,000; GE Healthcare). The bound antibodies were visualized with a LAS3000 imaging system (Fujifilm, Tokyo, Japan) using a Pierce enhanced chemiluminescence (ECL) western detection kit according to the manufacturer's instructions (Thermo Scientific). The intensity of each band was measured using Multi Gauge version 3.0 imaging software. The intensity of each *miR-634* target gene band was measured and normalized to the intensity of the β-actin band. The complete unedited images are represented in the Supplemental Information.

Measurement of Plasma AST and ALT Levels in Mice

AST and ALT levels were measured using an AST/ALT assay kit (Wako) on a Hitachi 7180 biochemical analyzer (Hitachi, Japan) in Oriental Yeast (Tokyo, Japan).

ELISA

ELISAs were performed to detect TNF-α, IFN-γ, and IL-6 protein levels in mouse serum. Quantitative analysis was performed using mouse-specific ELISA kits (TNF-α, KE10002; IFN-γ, KE10001; and IL-6, KE10007; Proteintech) according to the manufacturer's protocol.

RIP Microarray (RIP-Chip) and RIP-PCR Analyses

RIP was performed according to the manufacturer's instructions for the RiboCluster Profiler RIP assay kit for miRNA (MBL International). Cells were transfected with 10 nM *miR-NC* or *miR-634* and then lysed after 24 h. The lysates were precleared and incubated with AGO2 antibody-immobilized beads or normal rabbit IgG antibody-immobilized beads for RIP. Each bead complex was washed, and RNA was isolated. For gene expression analysis, RNA from AGO2-RIP and total RNA (input) were labeled and hybridized on the Agilent 8 × 60K array according to the manufacturer's instructions (Agilent Technologies). Each experiment was performed in duplicate, and the data were analyzed with GeneSpring software (Agilent Technologies). The 10,741 genes expressed in both *miR-NC*-overexpressing cells and *miR-634*-overexpressing cells were selected, and the enrichment (RIP/input) of these genes in the two groups of cells was calculated. Then, the fold change of enrichment (*miR-634*/*miR-NC*) was calculated. The fold change for the positive control *XIAP* (1.85), a *miR-634* target gene, was used as the cutoff value. The microarray data from this publication have been submitted to the GEO database (https://www.ncbi.nlm.nih.gov/geo/) and assigned the identifier GEO: GSE159093.

For RIP-PCR analysis, cDNA was synthesized from RNA isolated by AGO2-RIP or IgG-RIP and from total RNA (input), and real-time PCR was performed using primers within the *ASCT2* 3′ UTR (627 bp; forward, 5′-CTGCTGCGTCCCCACCGTGA-3′, and reverse, 5′-AACTACAGCCGCCAAAATA-3′) based on NCBI accession number GenBank: NM_005628.

Gene Expression Microarray Data Analysis

Three expression microarray datasets of GEO: GSE32628, GSE45216, and GSE98780 in cSCC samples were downloaded from GEO. There are 13 NSs, 14 AKs, and 15 cSCCs in GEO: GSE32628, 10 AKs and 30 cSCCs in GEO: GSE45216, and 16 non-sun-exposed (NSE) NSs, 20 sun-exposed (SE) NSs, and 18 AKs in GEO: GSE98780, respectively. In order to analyze a dataset combining GEO: GSE45216 and GSE98780, which are profiled using the same platform of Affymetrix Human Genome U133 Plus 2.0 Array, all CEL files of 94 total samples were assembled and processed together. Quality control (QC) and normalization were performed with GeneSpring software (Agilent Technologies, CA, USA). The available normalized expression values in $\log_2$ scale were used. Significant differential expressions were identified using the adjusted p value <0.05.

Proteomic Expression Analysis

Cells were transfected with 10 nM *miR-NC* or *miR-634* and lysed after 24 h. The iTRAQ-based proteomic expression analysis was performed by Integral Consulting. Each sample was digested with trypsin (AB Sciex), labeled with iTRAQ, combined, fractionated into six fractions by strong cation exchange chromatography (SCX), and desalted using a C18 column. Each peptide fraction was analyzed using a Q Exactive Plus (Thermo Fisher Scientific) coupled online with a capillary high-performance liquid chromatography (HPLC) system (EASY-nLC 1200, Thermo Fisher Scientific) to acquire tandem mass spectrometry (MS/MS) spectra. Proteome Discoverer (version 2.1, Thermo Fisher Scientific) was used to search the Swiss-Prot protein database and perform iTRAQ label-based quantification.

GO Analysis

GO analysis of differentially expressed genes was performed with DAVID (https://david.ncifcrf.gov/summary.jsp), and an enrichment score with p <0.05 was considered significant.

Immunofluorescence Analysis

For ASCT2 immunostaining, cells were fixed with 10% TCA for 15 min on ice, permeabilized with 0.1% Triton X-100 for 5 min,

blocked with PBS containing 0.01% Triton X-100 and 3% BSA for 1 h, and then incubated overnight with anti-ASCT2 antibodies (1:3,000 dilution). Bound antibodies were visualized using Alexa Fluor 488 anti-rabbit IgG antibody (1:2,000 dilution; Life Technologies), and coverslips were mounted in Vectashield containing DAPI (Vector Laboratories). Intracellular mitochondria were stained with 100 nmol/L MitoTracker Red CMXRos (Life Technologies) for 30 min at 37°C. After fixation with 10% TCA, coverslips were mounted in Vectashield containing DAPI (Vector Laboratories). Images were obtained by confocal fluorescence microscopy (Nikon).

Luciferase Reporter Assay

Luciferase reporter plasmids were constructed by inserting PCR products corresponding to the 3′ UTR of *ASCT2* into the *NheI/XhoI* sites downstream of the luciferase gene within the pmirGLO dual-luciferase miRNA target expression vector (Promega). The following primer sequences were used: *NheI*, forward, 5′-CTAGCTAGCCTGCTGCGTCCCCACCGTGA-3′; and *XhoI*, reverse, 5′-CCGCTCGAGCAACTACAGCCGCCAAAATA-3′. Site-specific mutagenesis was performed using the KOD Plus mutagenesis kit (Toyobo) and the following primers: forward, 5′-CCTCCTTATTTTGGCGGCTGTAGG-3′; and reverse, 5′-ATGGTGTTGTAACATCCGCTAG-3′. Cells were transfected with empty vector, WT *ASCT2* vector, or mutant (Mut) *ASCT2* vector and after 5 h with 20 nmol/L miRNA (*miR-NC* or *miR-634*). After 36 h of miRNA transfection, firefly and *Renilla* luciferase activity was measured using the dual-luciferase reporter assay system (Promega), and relative luciferase activity was calculated by normalizing firefly luciferase activity to *Renilla* luciferase activity (internal control).

Caspase-3/7 Activity Assay

Caspase-3/7 activity was determined with a Caspase-Glo 3/7 assay (Promega) according to the manufacturer's instructions. Luminescence was measured using a multiplate leader (BioTek).

Determination of Intracellular ROS Levels by Fluorescence-Activated Cell Sorting (FACS) Analysis

For the ROS detection assay, cells were incubated with 20 μM 2′,7′-dichlorofluorescein diacetate (DCFDA) for 30 min at 37°C with 5% CO_2. Fluorescence intensity was measured using an Accuri flow cytometer. For each analysis, the median fluorescence intensity was calculated using FlowJo software.

CI

Cells were treated with the indicated therapeutic combinations, and cell viability was measured using a CV staining assay. The Combination Index (CI) was calculated using CalcuSyn (Biosoft) according to the methods reported by Chou and Talley.[61] CI <1 indicates a synergistic drug-drug interaction.

Measurement of Metabolites

Metabolite levels per cells were measured by using the Lactate-Glo assay kit (Promega), Glutamine/Glutamate-Glo assay kit (Promega), *GSH/GSSG*-Glo assay kit (Promega), and luminescent ATP detection assay kit (Abcam) according to the manufacturers' instructions.

In Vivo Cy3-*miR-634* Permeability in Tumors

A 0.2% ointment incorporating Cy3-labeled *miR-634* (2 mg/mL) was formulated using ILTS, and Cy3-*miR-634* ointment was topically applied to subcutaneous A431 xenograft tumors in mice. At 1 h after ointment application, tumors were resected, fixed with 10% buffered formalin, infused in 10%–30% sucrose, and infiltrated with OCT. Tissues embedded in OCT were sectioned at 15-μm thickness and mounted in Vectashield containing DAPI (Vector Laboratories). Images were obtained by confocal fluorescence microscopy (Nikon).

DMBA/TPA-Induced Papilloma Mouse Model

Six-week-old male FVB/NJcl mice were purchased from Charles River Laboratories (Yokohama, Japan). To induce skin carcinogenesis, mice were treated with the two-stage carcinogenesis protocol, in which DMBA (Sigma-Aldrich) and TPA (Sigma-Aldrich) were used as the carcinogen and promoter, respectively. At 7–8 weeks of age, the mouse back was carefully shaved with an electric clipper. Two days after shaving, 200 μL of 487.5 μM DMBA dissolved in acetone was applied to the bare dorsal back skin. Seven days after the initial DMBA treatment, the mice were exposed to 400 μL of 81 μM TPA dissolved in acetone twice weekly for 10 weeks. Then, the mice were assessed for the presence of papilloma and divided into two groups. *miR-NC* ointment or *miR-634* ointment (20–40 μg miRNA) was topically applied to papillomas twice weekly, and the tumors were measured every 2 weeks. At 59 days after the first treatment, animals were sacrificed, and tumors were dissected. Papillomas were collected and frozen in liquid nitrogen.

Glutamine Uptake Assay

The culture medium was supplemented with 300 μg/ml L-glutamine-$^{15}N_2$ ($^{15}N_2$-Gln). After 15 min (for siRNA) or 2 h (for miRNAs), hydrophilic metabolites were extracted, and $^{15}N_2$-Gln levels were measured by liquid chromatography (LC)/MS analysis.

Statistical Analysis

Statistical significance was assessed by the two-tailed Student's t test or ANOVA (for multiple comparisons) using Prism version 5.04 (GraphPad, La Jolla, CA, USA). Results with $p \leq 0.05$ were considered statistically significant.

SUPPLEMENTAL INFORMATION

Supplemental Information can be found online at https://doi.org/10.1016/j.omto.2020.10.009.

ACKNOWLEDGMENTS

The authors thank Ayako Takahashi and Rumi Mori (Tokyo Medical and Dental University, Japan) for technical assistance. This work was supported in part by Grants-in-Aid for Scientific Research (18K06954 to J. Inoue; 16K14630 to J. Inazawa), a Grant-in-Aid for Scientific Research on Innovative Areas "Conquering cancer through NEO-dimensional systems understandings" (15H05908 to J. Inazawa)

from JSPS and MEXT, a research program of the Project for Cancer Research and Therapeutic Evolution (P-CREATE), and the Tailor-Made Medical Treatment with the BioBank Japan Project (BBJ) from the Japan Agency for Medical Research and Development (AMED). This study was supported by Nanken-Kyoten, TMDU.

AUTHOR CONTRIBUTIONS

J. Inoue and J. Inazawa contributed to the conception and design of study. J. Inoue and K.F. contributed to the acquisition and interpretation of data. H.H and K.K. formulated the ointments incorporating miRNAs. J. Inoue and J. Inazawa wrote the manuscript.

DECLARATION OF INTERESTS

The authors declare no competing interests.

REFERENCES

1. Kim, J.Y.S., Kozlow, J.H., Mittal, B., Moyer, J., Olenecki, T., and Rodgers, P.; Work Group; Invited Reviewers (2018). Guidelines of care for the management of cutaneous squamous cell carcinoma. J. Am. Acad. Dermatol. *78*, 560–578.
2. Alam, M., and Ratner, D. (2001). Cutaneous squamous-cell carcinoma. N. Engl. J. Med. *344*, 975–983.
3. Lewis, C.M., Glisson, B.S., Feng, L., Wan, F., Tang, X., Wistuba, I.I., El-Naggar, A.K., Rosenthal, D.I., Chambers, M.S., Lustig, R.A., and Weber, R.S. (2012). A phase II study of gefitinib for aggressive cutaneous squamous cell carcinoma of the head and neck. Clin. Cancer Res. *18*, 1435–1446.
4. Nehal, K.S., and Bichakjian, C.K. (2018). Update on keratinocyte carcinomas. N. Engl. J. Med. *379*, 363–374.
5. Fayne, R., Nanda, S., Nichols, A., and Shen, J. (2020). Combination topical chemotherapy for the treatment of an invasive cutaneous squamous cell carcinoma. J. Drugs Dermatol. *19*, 202–204.
6. Jansen, M.H.E., Mosterd, K., Arits, A.H.M.M., Roozeboom, M.H., Sommer, A., Essers, B.A.B., van Pelt, H.P.A., Quaedvlieg, P.J.F., Steijlen, P.M., Nelemans, P.J., and Kelleners-Smeets, N.W.J. (2018). Five-year results of a randomized controlled trial comparing effectiveness of photodynamic therapy, topical imiquimod, and topical 5-fluorouracil in patients with superficial basal cell carcinoma. J. Invest. Dermatol. *138*, 527–533.
7. Chitwood, K., Etzkorn, J., and Cohen, G. (2013). Topical and intralesional treatment of nonmelanoma skin cancer: efficacy and cost comparisons. Dermatol. Surg. *39*, 1306–1316.
8. Collins, A., Savas, J., and Doerfler, L. (2019). Nonsurgical treatments for nonmelanoma skin cancer. Dermatol. Clin. *37*, 435–441.
9. Bath-Hextall, F., Ozolins, M., Armstrong, S.J., Colver, G.B., Perkins, W., Miller, P.S., and Williams, H.C.; Surgery versus Imiquimod for Nodular Superficial basal cell carcinoma (SINS) study group (2014). Surgical excision versus imiquimod 5% cream for nodular and superficial basal-cell carcinoma (SINS): a multicentre, non-inferiority, randomised controlled trial. Lancet Oncol. *15*, 96–105.
10. Cañueto, J., Cardeñoso, E., García, J.L., Santos-Briz, Á., Castellanos-Martín, A., Fernández-López, E., Blanco Gómez, A., Pérez-Losada, J., and Román-Curto, C. (2017). Epidermal growth factor receptor expression is associated with poor outcome in cutaneous squamous cell carcinoma. Br. J. Dermatol. *176*, 1279–1287.
11. Ratushny, V., Gober, M.D., Hick, R., Ridky, T.W., and Seykora, J.T. (2012). From keratinocyte to cancer: the pathogenesis and modeling of cutaneous squamous cell carcinoma. J. Clin. Invest. *122*, 464–472.
12. Di Nardo, L., Pellegrini, C., Di Stefani, A., Del Regno, L., Sollena, P., Piccerillo, A., Longo, C., Garbe, C., Fargnoli, M.C., and Peris, K. (2020). Molecular genetics of cutaneous squamous cell carcinoma: perspective for treatment strategies. J. Eur. Acad. Dermatol. Venereol. *34*, 932–941.
13. Maubec, E., Petrow, P., Scheer-Senyarich, I., Duvillard, P., Lacroix, L., Gelly, J., Certain, A., Duval, X., Crickx, B., Buffard, V., et al. (2011). Phase II study of cetuximab as first-line single-drug therapy in patients with unresectable squamous cell carcinoma of the skin. J. Clin. Oncol. *29*, 3419–3426.
14. William, W.N., Jr., Feng, L., Ferrarotto, R., Ginsberg, L., Kies, M., Lippman, S., Glisson, B., and Kim, E.S. (2017). Gefitinib for patients with incurable cutaneous squamous cell carcinoma: a single-arm phase II clinical trial. J. Am. Acad. Dermatol. *77*, 1110–1113.e2.
15. Gold, K.A., Kies, M.S., William, W.N., Jr., Johnson, F.M., Lee, J.J., and Glisson, B.S. (2018). Erlotinib in the treatment of recurrent or metastatic cutaneous squamous cell carcinoma: a single-arm phase 2 clinical trial. Cancer *124*, 2169–2173.
16. Okamoto, K., Okamoto, I., Okamoto, W., Tanaka, K., Takezawa, K., Kuwata, K., Yamaguchi, H., Nishio, K., and Nakagawa, K. (2010). Role of survivin in EGFR inhibitor-induced apoptosis in non-small cell lung cancers positive for EGFR mutations. Cancer Res. *70*, 10402–10410.
17. Park, S.H., Kim, J.H., Ko, E., Kim, J.Y., Park, M.J., Kim, M.J., Seo, H., Li, S., and Lee, J.Y. (2018). Resistance to gefitinib and cross-resistance to irreversible EGFR-TKIs mediated by disruption of the Keap1-Nrf2 pathway in human lung cancer cells. FASEB J. *32*, fj201800011R.
18. Ou, C., Liu, H., Ding, Z., and Zhou, L. (2019). Chloroquine promotes gefitinib-induced apoptosis by inhibiting protective autophagy in cutaneous squamous cell carcinoma. Mol. Med. Rep. *20*, 4855–4866.
19. Wang, J., Wang, C., Hu, X., Yu, C., Zhou, L., Ding, Z., and Zhou, M. (2019). Gefitinib-mediated apoptosis is enhanced via inhibition of autophagy by chloroquine diphosphate in cutaneous squamous cell carcinoma cells. Oncol. Lett. *18*, 368–374.
20. Wang, L., Peng, W., Wu, T., Deng, P., and Zhao, Y.L. (2018). Increased glutamine anabolism sensitizes non-small cell lung cancer to gefitinib treatment. Cell Death Discov. *4*, 24.
21. Momcilovic, M., Bailey, S.T., Lee, J.T., Fishbein, M.C., Magyar, C., Braas, D., Graeber, T., Jackson, N.J., Czernin, J., Emberley, E., et al. (2017). Targeted Inhibition of EGFR and glutaminase induces metabolic crisis in EGFR mutant lung cancer. Cell Rep. *18*, 601–610.
22. Gebert, L.F.R., and MacRae, I.J. (2019). Regulation of microRNA function in animals. Nat. Rev. Mol. Cell Biol. *20*, 21–37.
23. O'Brien, J., Hayder, H., Zayed, Y., and Peng, C. (2018). Overview of microRNA biogenesis, mechanisms of actions, and circulation. Front. Endocrinol. (Lausanne) *9*, 402.
24. Mollaei, H., Safaralizadeh, R., and Rostami, Z. (2019). MicroRNA replacement therapy in cancer. J. Cell. Physiol. *234*, 12369–12384.
25. Rupaimoole, R., and Slack, F.J. (2017). MicroRNA therapeutics: towards a new era for the management of cancer and other diseases. Nat. Rev. Drug Discov. *16*, 203–222.
26. Kozaki, K., and Inazawa, J. (2012). Tumor-suppressive microRNA silenced by tumor-specific DNA hypermethylation in cancer cells. Cancer Sci. *103*, 837–845.
27. Tsuruta, T., Kozaki, K., Uesugi, A., Furuta, M., Hirasawa, A., Imoto, I., Susumu, N., Aoki, D., and Inazawa, J. (2011). miR-152 is a tumor suppressor microRNA that is silenced by DNA hypermethylation in endometrial cancer. Cancer Res. *71*, 6450–6462.
28. Uesugi, A., Kozaki, K., Tsuruta, T., Furuta, M., Morita, K., Imoto, I., Omura, K., and Inazawa, J. (2011). The tumor suppressive microRNA miR-218 targets the mTOR component Rictor and inhibits AKT phosphorylation in oral cancer. Cancer Res. *71*, 5765–5778.
29. Fujiwara, N., Inoue, J., Kawano, T., Tanimoto, K., Kozaki, K., and Inazawa, J. (2015). miR-634 activates the mitochondrial apoptosis pathway and enhances chemotherapy-induced cytotoxicity. Cancer Res. *75*, 3890–3901.
30. Endo, H., Muramatsu, T., Furuta, M., Uzawa, N., Pimkhaokham, A., Amagasa, T., Inazawa, J., and Kozaki, K. (2013). Potential of tumor-suppressive *miR-596* targeting *LGALS3BP* as a therapeutic agent in oral cancer. Carcinogenesis *34*, 560–569.
31. Harazono, Y., Muramatsu, T., Endo, H., Uzawa, N., Kawano, T., Harada, K., Inazawa, J., and Kozaki, K. (2013). *miR-655* is an EMT-suppressive microRNA targeting *ZEB1* and *TGFBR2*. PLoS ONE *8*, e62757.
32. Yamamoto, S., Inoue, J., Kawano, T., Kozaki, K., Omura, K., and Inazawa, J. (2014). The impact of miRNA-based molecular diagnostics and treatment of NRF2-stabilized tumors. Mol. Cancer Res. *12*, 58–68.

33. Tonouchi, E., Gen, Y., Muramatsu, T., Hiramoto, H., Tanimoto, K., Inoue, J., and Inazawa, J. (2018). *miR-3140* suppresses tumor cell growth by targeting *BRD4* via its coding sequence and downregulates the BRD4-NUT fusion oncoprotein. Sci. Rep. *8*, 4482.

34. Takagawa, Y., Gen, Y., Muramatsu, T., Tanimoto, K., Inoue, J., Harada, H., and Inazawa, J. (2020). *miR-1293*, a candidate for miRNA-based cancer therapeutics, simultaneously targets *BRD4* and the DNA repair pathway. Mol. Ther. *28*, 1494–1505.

35. Gokita, K., Inoue, J., Ishihara, H., Kojima, K., and Inazawa, J. (2020). Therapeutic potential of LNP-mediated delivery of miR-634 for cancer therapy. Mol. Ther. Nucleic Acids *19*, 330–338.

36. Kabeya, Y., Mizushima, N., Ueno, T., Yamamoto, A., Kirisako, T., Noda, T., Kominami, E., Ohsumi, Y., and Yoshimori, T. (2000). LC3, a mammalian homologue of yeast Apg8p, is localized in autophagosome membranes after processing. EMBO J. *19*, 5720–5728.

37. Klionsky, D.J., Abdalla, F.C., Abeliovich, H., Abraham, R.T., Acevedo-Arozena, A., Adeli, K., Agholme, L., Agnello, M., Agostinis, P., Aguirre-Ghiso, J.A., et al. (2012). Guidelines for the use and interpretation of assays for monitoring autophagy. Autophagy *8*, 445–544.

38. Handa, Y., Ugajin, T., Igawa, K., Hamamoto, H., Kobayashi, K., Komatsuno, T., Yamamoto, T., Kawahara, K., and Yokozeki, H. (2019). STAT6 decoy oligodeoxynucleotide (ODN)-containing ointment more potently inhibits mouse skin inflammation when formulated with ionic liquid technology than as a traditional Vaseline ointment. Allergol. Int. *68*, 380–382.

39. Furusawa, E., Ohno, T., Nagai, S., Noda, T., Komiyama, T., Kobayashi, K., Hamamoto, H., Miyashin, M., Yokozeki, H., and Azuma, M. (2019). Silencing of PD-L2/B7-DC by topical application of small interfering RNA inhibits elicitation of contact hypersensitivity. J. Invest. Dermatol. *139*, 2164–2173.e1.

40. Kobayashi, H., and Tomari, Y. (2016). RISC assembly: coordination between small RNAs and Argonaute proteins. Biochim. Biophys. Acta *1859*, 71–81.

41. Keene, J.D., Komisarow, J.M., and Friedersdorf, M.B. (2006). RIP-chip: the isolation and identification of mRNAs, microRNAs and protein components of ribonucleoprotein complexes from cell extracts. Nat. Protoc. *1*, 302–307.

42. Liu, Y., Zhao, T., Li, Z., Wang, L., Yuan, S., and Sun, L. (2018). The role of ASCT2 in cancer: a review. Eur. J. Pharmacol. *837*, 81–87.

43. Makinoshima, H., Takita, M., Matsumoto, S., Yagishita, A., Owada, S., Esumi, H., and Tsuchihara, K. (2014). Epidermal growth factor receptor (EGFR) signaling regulates global metabolic pathways in EGFR-mutated lung adenocarcinoma. J. Biol. Chem. *289*, 20813–20823.

44. Su, H., Bodenstein, C., Dumont, R.A., Seimbille, Y., Dubinett, S., Phelps, M.E., Herschman, H., Czernin, J., and Weber, W. (2006). Monitoring tumor glucose utilization by positron emission tomography for the prediction of treatment response to epidermal growth factor receptor kinase inhibitors. Clin. Cancer Res. *12*, 5659–5667.

45. Diaz-Vivancos, P., de Simone, A., Kiddle, G., and Foyer, C.H. (2015). Glutathione—linking cell proliferation to oxidative stress. Free Radic. Biol. Med. *89*, 1154–1164.

46. Frazier, K.S. (2015). Antisense oligonucleotide therapies: the promise and the challenges from a toxicologic pathologist's perspective. Toxicol. Pathol. *43*, 78–89.

47. Lindow, M., Vornlocher, H.P., Riley, D., Kornbrust, D.J., Burchard, J., Whiteley, L.O., Kamens, J., Thompson, J.D., Nochur, S., Younis, H., et al. (2012). Assessing unintended hybridization-induced biological effects of oligonucleotides. Nat. Biotechnol. *30*, 920–923.

48. Yoshida, T., Naito, Y., Sasaki, K., Uchida, E., Sato, Y., Naito, M., Kawanishi, T., Obika, S., and Inoue, T. (2018). Estimated number of off-target candidate sites for antisense oligonucleotides in human mRNA sequences. Genes Cells *23*, 448–455.

49. Meng, Z., and Lu, M. (2017). RNA interference-induced innate immunity, off-target effect, or immune adjuvant? Front. Immunol. *8*, 331.

50. Hong, D.S., Kang, Y.K., Borad, M., Sachdev, J., Ejadi, S., Lim, H.Y., Brenner, A.J., Park, K., Lee, J.L., Kim, T.Y., et al. (2020). Phase 1 study of MRX34, a liposomal miR-34a mimic, in patients with advanced solid tumours. Br. J. Cancer *122*, 1630–1637.

51. Koppenol, W.H., Bounds, P.L., and Dang, C.V. (2011). Otto Warburg's contributions to current concepts of cancer metabolism. Nat. Rev. Cancer *11*, 325–337.

52. Gellrich, F.F., Hüning, S., Beissert, S., Eigentler, T., Stockfleth, E., Gutzmer, R., and Meier, F. (2019). Medical treatment of advanced cutaneous squamous-cell carcinoma. J. Eur. Acad. Dermatol. Venereol. *33* (*Suppl 8*), 38–43.

53. Migden, M.R., Rischin, D., Schmults, C.D., Guminski, A., Hauschild, A., Lewis, K.D., Chung, C.H., Hernandez-Aya, L., Lim, A.M., Chang, A.L.S., et al. (2018). PD-1 blockade with cemiplimab in advanced cutaneous squamous-cell carcinoma. N. Engl. J. Med. *379*, 341–351.

54. Guminski, A.D., Lim, A.M.L., Khushalani, N.I., Schmults, C.D., Hernandez-Aya, L.F., Modi, B., Dunn, L., Hughes, B.G.M., Chang, A.L.S., Hauschild, A., et al. (2019). Phase 2 study of cemiplimab, a human monoclonal anti-PD-1, in patients (pts) with metastatic cutaneous squamous cell carcinoma (mCSCC; group 1): 12-month follow-up. J. Clin. Oncol. *37* (15 Suppl), 9526.

55. Migden, M.R., Khushalani, N.I., Chang, A.L.S., Rishin, D., Schmults, C.D., Hernandez-Aya, L.F., Meier, F.E., Schadendorf, Guminski, A.D., Hauschild, A., et al. (2019). Primary analysis of phase 2 results of cemiplimab, a human monoclonal anti-PD-1, in patients (pts) with locally advanced cutaneous squamous cell carcinoma (laCSCC). J. Clin. Oncol. *37* (15 Suppl), 6015.

56. Kittai, A.S., Oldham, H., Cetnar, J., and Taylor, M. (2017). Immune checkpoint inhibitors in organ transplant patients. J. Immunother. *40*, 277–281.

57. Miwa, Y., Hamamoto, H., and Ishida, T. (2016). Lidocaine self-sacrificially improves the skin permeation of the acidic and poorly water-soluble drug etodolac via its transformation into an ionic liquid. Eur. J. Pharm. Biopharm. *102*, 92–100.

58. Kubota, K., Shibata, A., and Yamaguchi, T. (2016). The molecular assembly of the ionic liquid/aliphatic carboxylic acid/aliphatic amine as effective and safety transdermal permeation enhancers. Eur. J. Pharm. Sci. *86*, 75–83.

59. Davis, S., Lollo, B., Freier, S., and Esau, C. (2006). Improved targeting of miRNA with antisense oligonucleotides. Nucleic Acids Res. *34*, 2294–2304.

60. Lennox, K.A., and Behlke, M.A. (2011). Chemical modification and design of anti-miRNA oligonucleotides. Gene Ther. *18*, 1111–1120.

61. Chou, T.C. (2010). Drug combination studies and their synergy quantification using the Chou-Talalay method. Cancer Res. *70*, 440–446.

OMTO, Volume *19*

Supplemental Information

Improving the Efficacy of EGFR Inhibitors by Topical Treatment of Cutaneous Squamous Cell Carcinoma with *miR-634* Ointment

Jun Inoue, Kyoko Fujiwara, Hidetoshi Hamamoto, Katsunori Kobayashi, and Johji Inazawa

Figure S1

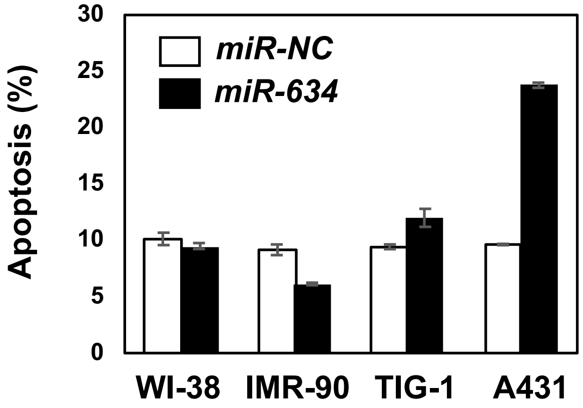

FACS analysis of the apoptotic cell population

FACS analysis was performed at 3 days after transfection. Cells were collected and stained with Annexin V and propidium iodide (PI). Cell population analysis was performed using an Accuri Flow Cytometer. The percentages of apoptotic cells are indicated in each graph. Error bars indicate SD of triplicate experiments.

Figure S2

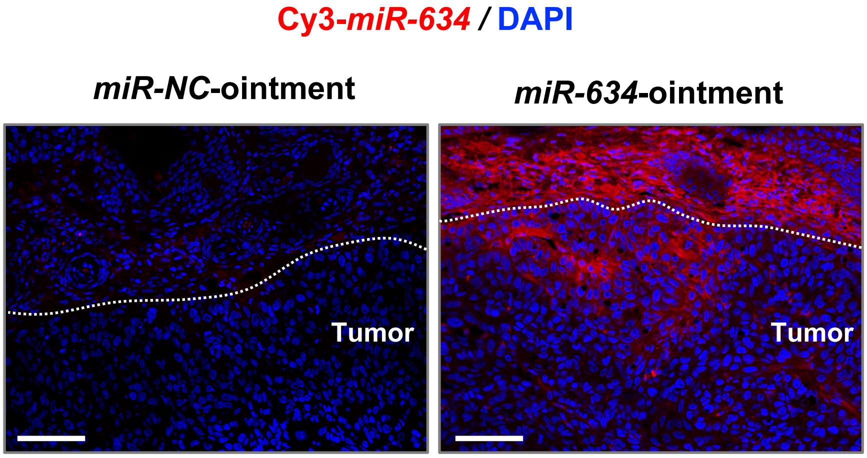

Fluorescence images of frozen subcutaneous tumor sections.
Cy-3-labelled *miR-634* ointment was topically applied to subcutaneous A431 xenograft tumors in mice. At 1 h after application, the tumors were resected, and frozen sections were prepared. Dotted lines indicate the border between tumor tissue and mouse skin. Cy3-*miR-634*, red. DAPI, blue. Scale bar, 100 μm.

Figure S3

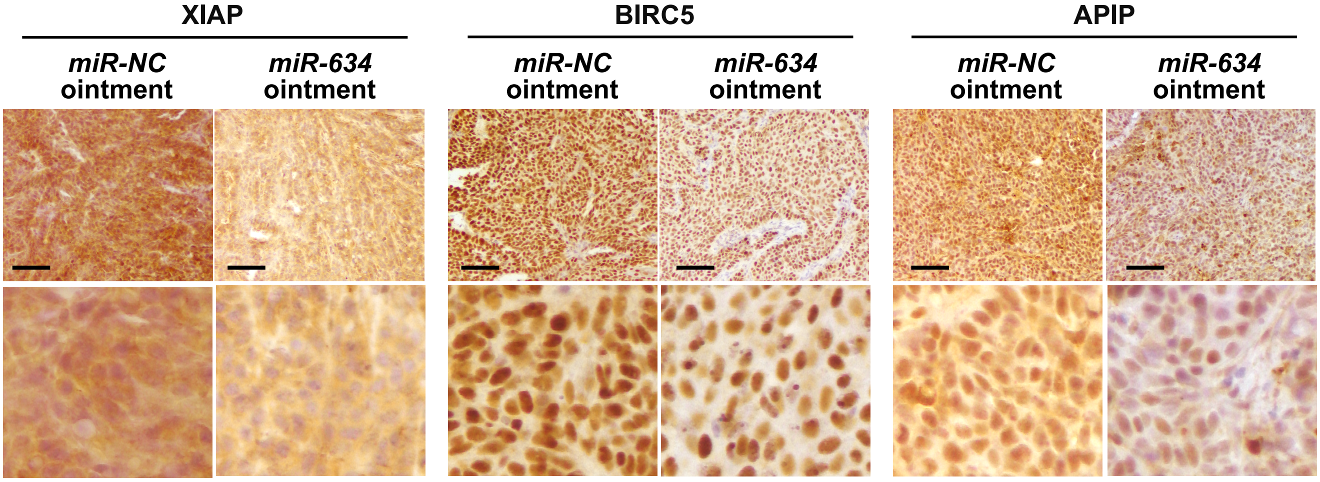

Immunohistochemistry (IHC) analysis of XIAP, BIRC5, and APIP in tumors from mice treated with *miR-NC* ointment or *miR-634* ointment.
Lower panel; enlarged images. Bar; 100 μm.

Figure S4

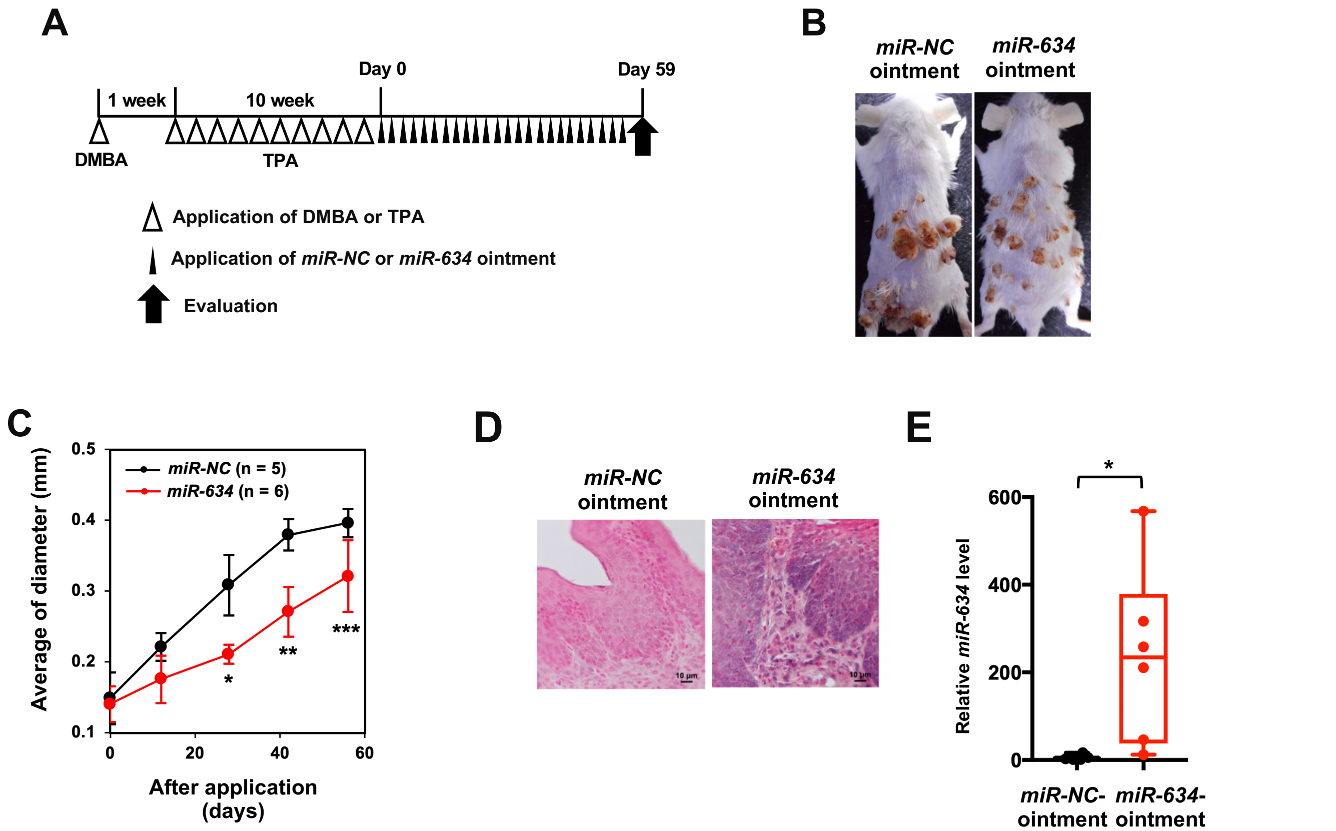

Antitumor effect of *miR-634* ointment in the DMPA/TPA-induced skin papilloma mouse model

A. Experimental schedule for the induction of papilloma and treatment. *miR-NC* ointment or *miR-634* ointment was topically applied twice per week. At 59 days after the first application, the mice were sacrificed, and papillomas were evaluated.
B. Representative images of papilloma after 59 day of treatment.
C. The average diameter was measured in mice treated with *miR-NC* ointment (n = 5) or *miR-634* ointment (n = 6). Error bars indicate the SD. Data are presented as mean ± SD. *P*-values were calculated using the two-sided Student's *t*-test (**p* = 0.0005 at 28 days, ***p* = 0.00019 at 42 days, and ****p* = 0.0013 at 59 days).
D. *In situ* hybridization (ISH) analysis of *miR-634* in resected papilloma. Representative ISH images in papillomas from mice treated with *miR-NC* ointment or *miR-634* ointment. The *miR-634*-specific probe appears purple in the cytoplasm, and the nucleus was counterstained with nuclear fast red. Scale bars, 10 μm.
E. qRT-PCR expression analysis of *miR-634* in resected papillomas. *miR-634* expression levels in papillomas from mice treated with *miR-NC* ointment (n = 5) or *miR-634* ointment (n = 6) were measured by qRT-PCR, and the results are presented in the box plot. *P*-values were calculated using the two-sided Student's *t*-test (**p* = 0.019).

Figure S5

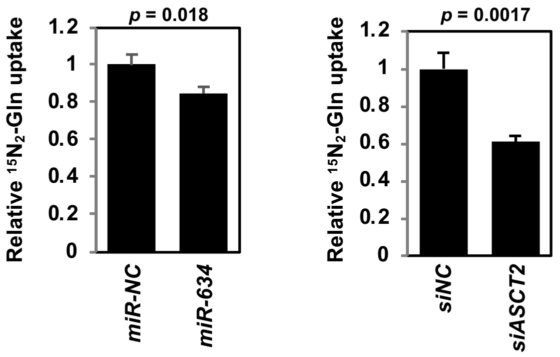

Glutamine uptake assays in *miR-634*-overexpressing cells and *ASCT2*-inhibited cells

A431 cells were transfected with *miR-NC* or *miR-634* (left) and siRNA-*NC* (*siNC*) or siRNA-*ASCT2* (*siASCT2*). After 3 days of transfection, $^{15}N_2$-labelled glutamine was added to the medium, and metabolites were extracted after 2 h (for miRNAs) or 15 min (for siRNAs). Intracellular $^{15}N_2$-labelled glutamine levels per cells were measured using LC/MS. Data are presented as mean ± SD. *P*-values were calculated using the two-sided Student's *t*-test.

Figure S6

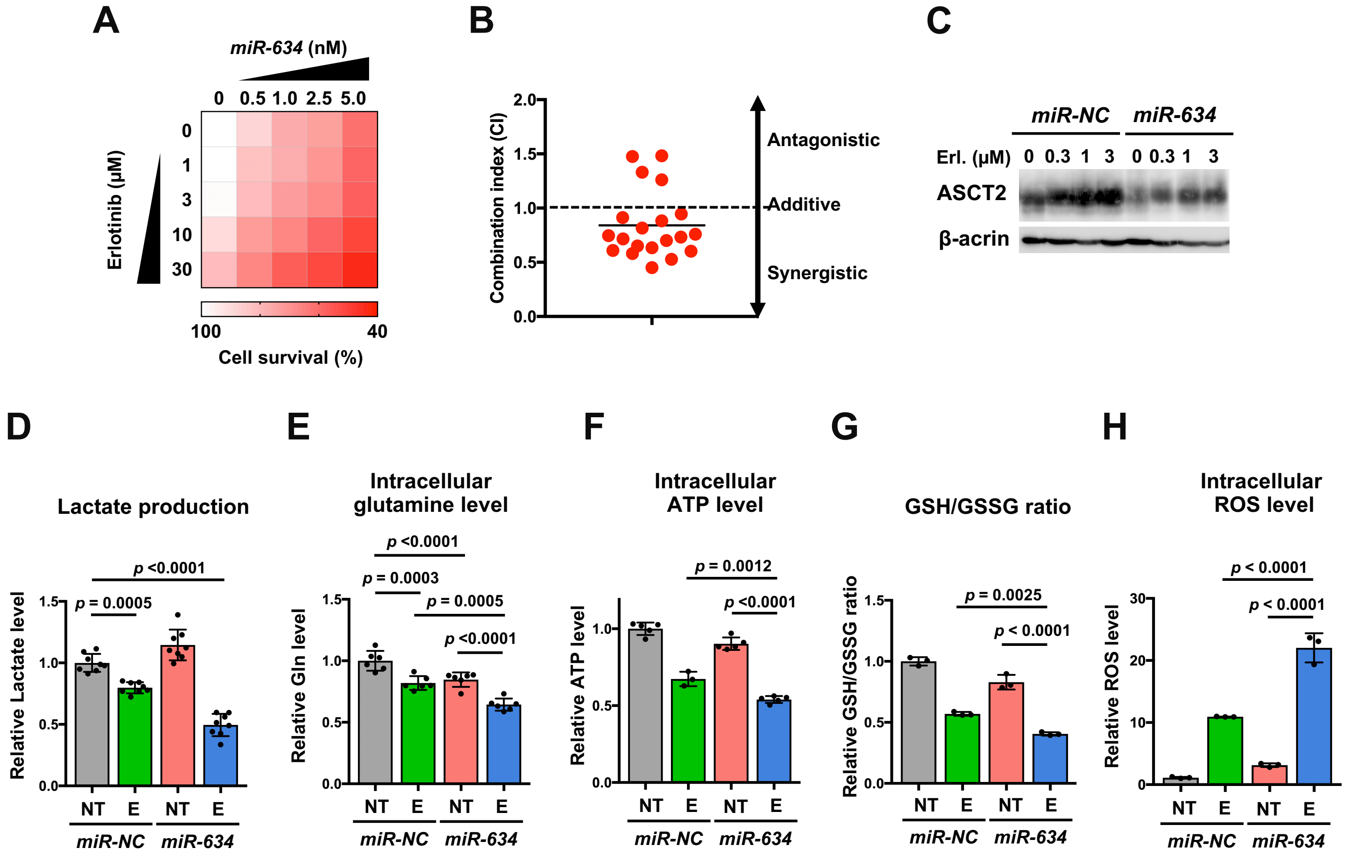

Augmentation of erlotinib-induced cytotoxicity by *miR-634*

A. Cell survival assay. A431 cells were transfected with increasing doses of *miR-634* (1.0 to 10 nM) of *miR-634* and simultaneously treated with erlotinib (1 to 30 μM). After 24 h of erlotinib treatment, the cell survival rate was assessed by crystal violet (CV) staining, and the results are reported as the relative rate compared with non-transfected and non-treated cells.

B. Combination index for A431 cells treated with erlotinib and *miR-634*.

C. Western blotting analysis of ASCT2 and apoptosis markers. Cell lysates were separated by SDS-PAGE and immunoreacted with the indicated antibodies.

D-H. Metabolite analysis of A431 cells treated with erlotinib (E) and/or *miR-634*. A431 cells were transfected with 10 nM of *miR-NC* or *miR-634* and then treated with erlotinib (3 μM) 24 h later. After 24 h of erlotinib treatment, lactate production (d), intracellular glutamine levels (e), intracellular ATP levels (f), the GSH/GSSG ratio (g), and intracellular ROS levels (h) were measured as described in the materials and methods. Data are presented as mean ± SD. *P*-values were calculated using two-way ANOVA.

Figure S7

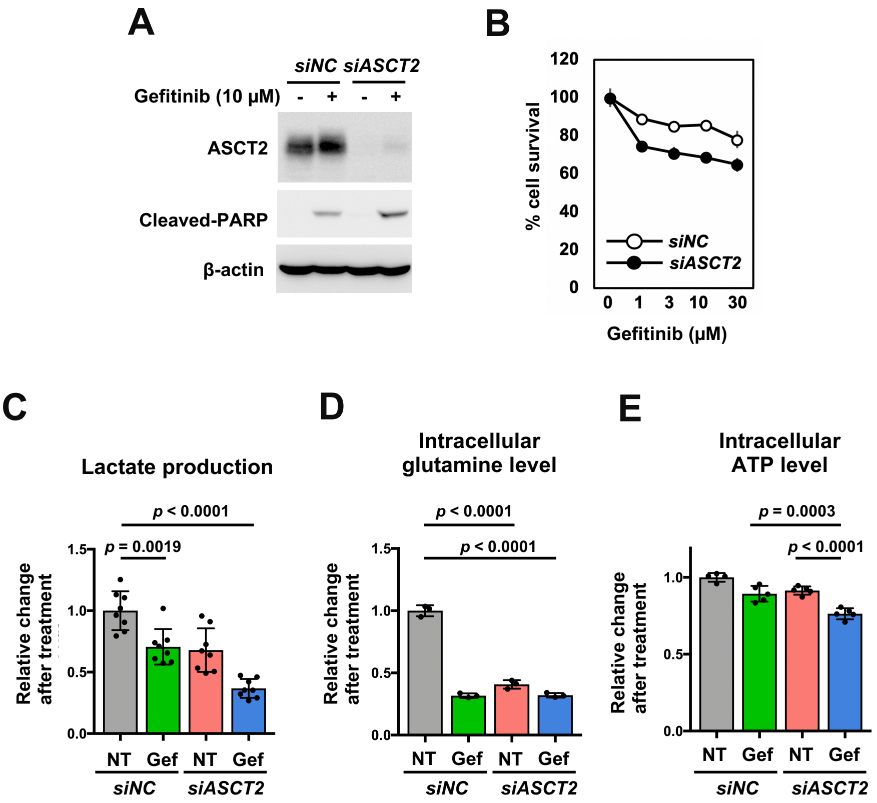

Augmentation of gefitinib-induced cytotoxicity by *ASCT2* knockdown

A. Western blotting analysis of ASCT2 and cleaved PARP. Cell lysates were separated by SDS-PAGE and immunoreacted with the indicated antibodies. Relative expression levels of target genes.

B. Cell survival assay. A431 cells were transfected with 10 nM NC siRNA (*siNC*) or *ASCT2* siRNA (*siASCT2*) and simultaneously treated with gefitinib (1 to 30 µM). After 24 h of gefitinib treatment, the cell survival rate was assessed by crystal violet (CV) staining, and the results are reported as the relative rate compared with non-transfected and non-treated cells.

C-E. Metabolite analysis of A431 cells treated with gefitinib (Gef) and/or *miR-634*. A431 cells were transfected with 10 nM *miR-NC* or *miR-634* and then treated with gefitinib (10 µM) 24 h later. After 24 h of gefitinib treatment, lactate production (c), intracellular glutamine levels (d), and ATP production (e) were measured as described in the materials and methods. Data are presented as mean ± SD. *P*-values were calculated using two-way ANOVA.

Table S1
List of 428 genes downregulated at the protein level in miR-634-overexpressing cells

Table S2
List of 110 genes commonly overlapped from two analyses that were defined as genes directly targeted by miR-634 in A431 cells

Table S3
GO analysis of 110 candidate genes for miR-634 targets

Complete unedited images for western blots

Figure 1B

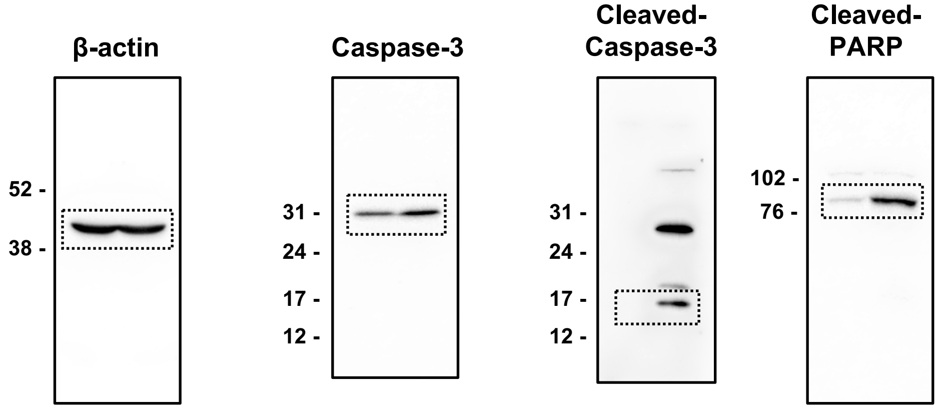

Figure 1D

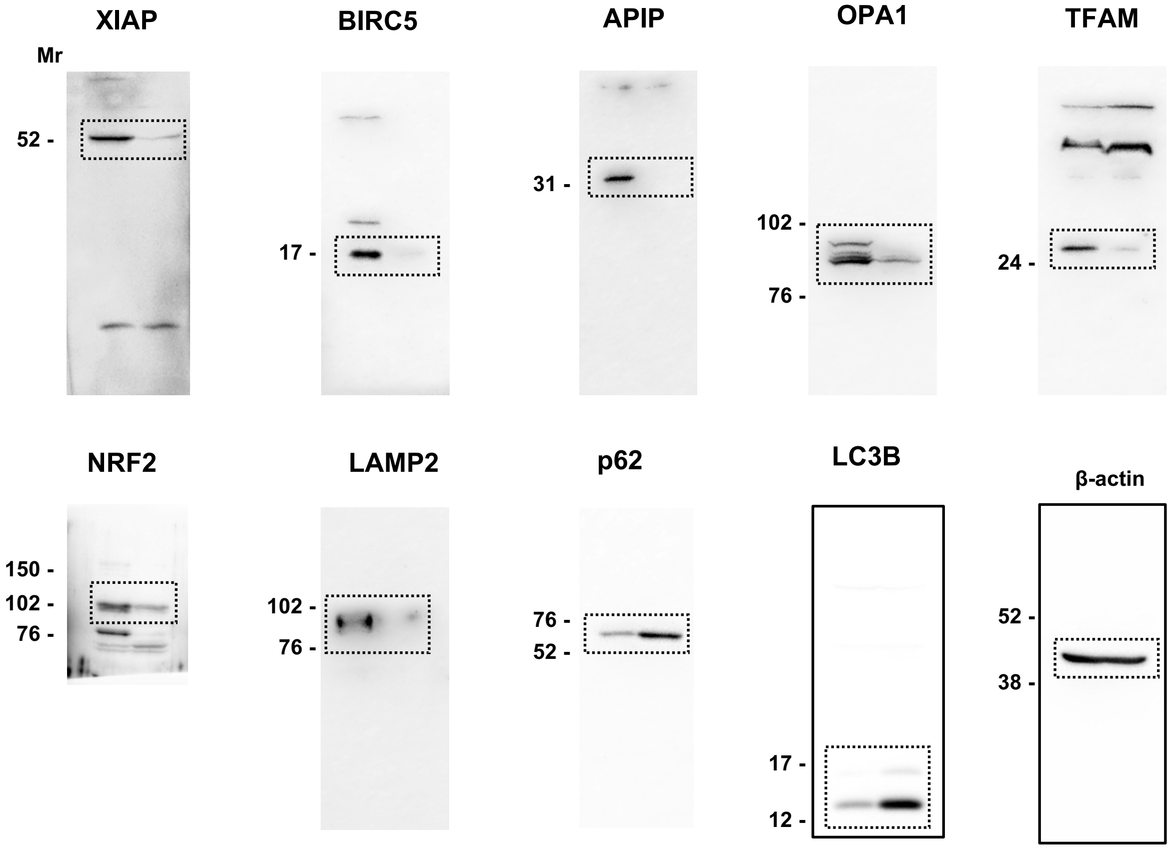

Complete unedited images for western blots (contined)

Figure 1I

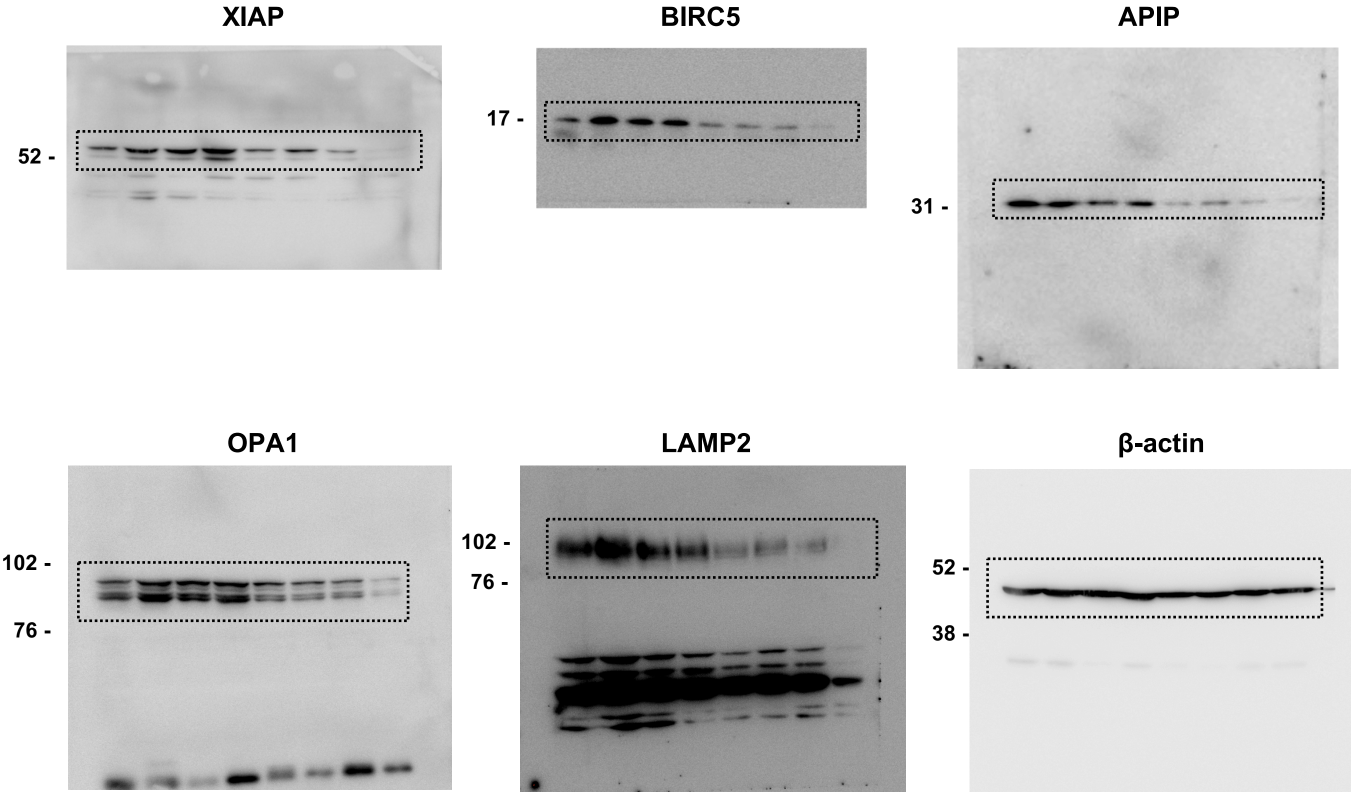

Figure 3

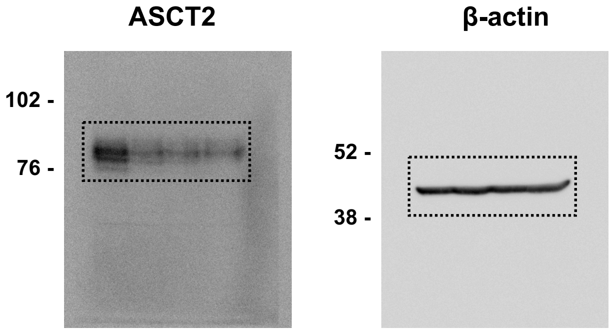

Figure 3F

Figure 3F

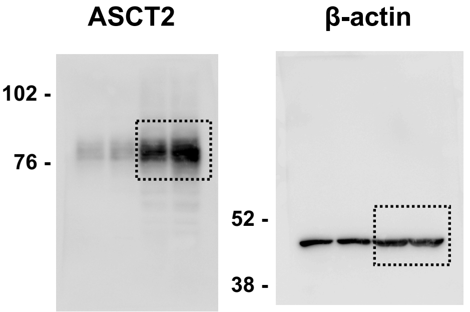

Figure 3G

Figure 3G

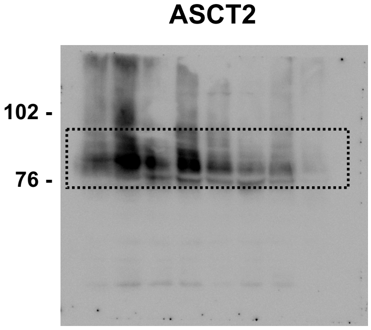

Figure 3I

Complete unedited images for western blots (contined)

Figure 4D

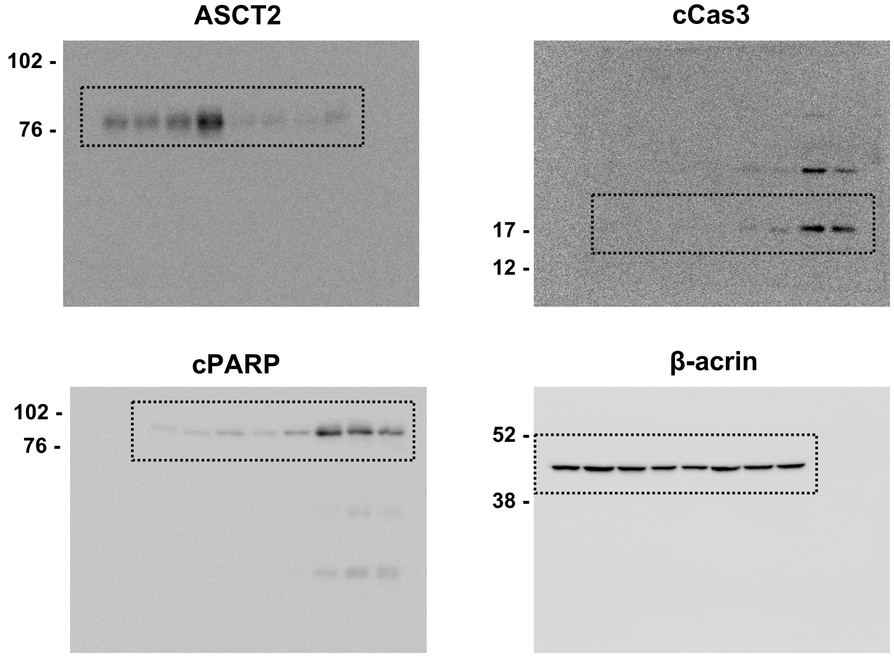

Figure S6C

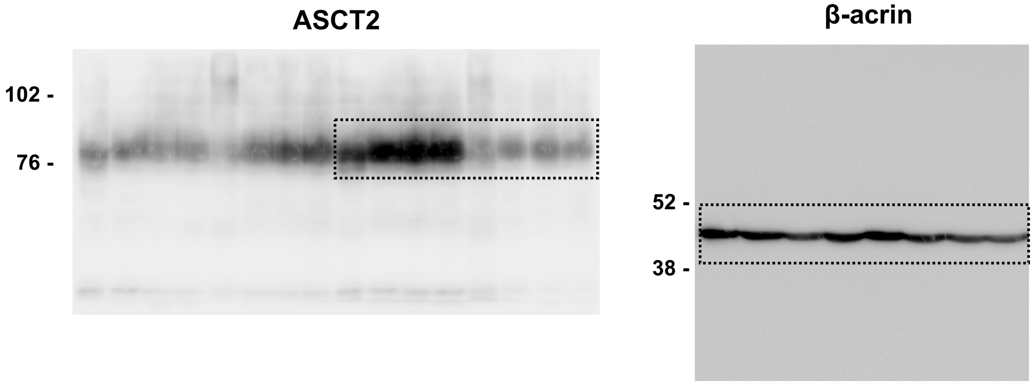

Figure S7A

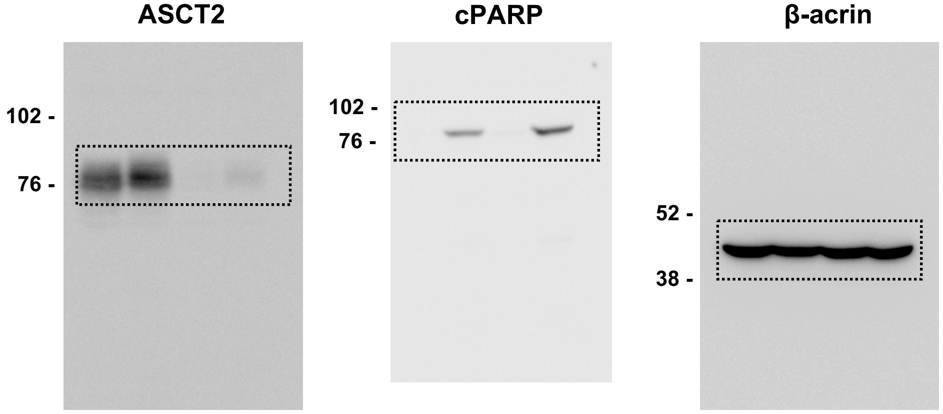

Supplement: Document S3. Article plus Supplemental Information [file mmc3.pdf]
